# An fMRI study of scientists with a Ph.D. in physics confronted with naive ideas in science

**DOI:** 10.1038/s41539-021-00091-x

**Published:** 2021-05-11

**Authors:** Geneviève Allaire-Duquette, Lorie-Marlène Brault Foisy, Patrice Potvin, Martin Riopel, Marilyne Larose, Steve Masson

**Affiliations:** Université du Québec à Montréal, Montréal, QC Canada

**Keywords:** Education, Cognitive control

## Abstract

A central challenge in developing conceptual understanding in science is overcoming naive ideas that contradict the content of science curricula. Neuroimaging studies reveal that high school and university students activate frontal brain areas associated with inhibitory control to overcome naive ideas in science, probably because they persist despite scientific training. However, no neuroimaging study has yet explored how persistent naive ideas in science are. Here, we report brain activations of 25 scientists with a Ph.D. in physics assessing the scientific value of naive ideas in science. Results show that scientists are slower and have lower accuracy when judging the scientific value of naive ideas compared to matched control ideas. fMRI data reveals that a network of frontal brain regions is more activated when judging naive ideas. Results suggest that naive ideas are likely to persist, even after completing a Ph.D. Advanced experts may still rely on high order executive functions like inhibitory control to overcome naive ideas when the context requires it.

## Introduction

Over the course of their lives, humans hold to many naive ideas or misconceptions about how nature works. ﻿Contrary to what one might have thought, these naive ideas are often far from being irrational or senseless and can even be useful in everyday living. However, some naive ideas have proved to be a “pain in the neck” for science educators, since they are often incompatible with the content of science curricula. They are also sometimes very resistant to change^1–5^. It is, for instance, naively and widely believed that summer is the warmest season because the Earth is the closest to the Sun^6,7^. This naive idea that warmer means closer is quite useful in day-to-day life and is probably reinforced by common hands-on experiences, such as approaching a hand from the stovetop or a campfire and getting a warmer sensation. Hence, when teaching seasons on Earth, educators can be confronted with a prevailing naive idea that competes with the scientific reason according to which seasons are the result of a change in the pattern of the Sun’s apparent motion, which results in changes in the intensity of the Sun’s rays and changes in the length of day.

One important goal of science education is therefore to encourage the conceptual change process, a learning pathway from naive or pre‐instructional ideas to the scientific ideas^8^. However, conceptual change is often very hard to accomplish, and students frequently revert to naive ideas despite carefully planned teaching^9–13^. Recent contributions to conceptual change literature highlight the difficulty to cope with the coexistence of naive and scientific ideas within an individual’s mind. Naive ideas more than scientific ones rely on fast and effective cognitive resources that are deeply anchored in human intuitions or heuristics and reinforced in real‐life contexts^14–21^. Indeed, behavioral studies show a tendency to endorse naive ideas in speeded response conditions^22,23^ and observe increased response time to process problems that trigger naive ideas of science^24–26^. Altogether, these findings are interpreted as evidence of an increased demand on executive control to allow resistance to distractors and interferences^27^. Hence, conceptual change is thought to be contingent upon the capacity to suppress or inhibit dominant naive ideas to allow the expression of less prevalent scientific ones^28^.

Behavioral studies explored the processing of naive ideas in experts with statement-verification tasks. Results suggest that mature scientific knowledge doesn’t involve radically overwriting or erasing naive ideas. Experts in biology exhibit a confusion similar to the one of children when confronted with the naive ideas that moving artifacts are alive^29^. Scientists from top-ranked American universities demonstrate a default bias toward naive ideas related to teleological explanations (e.g., cows have udders in order to allow farmers to milk them) despite their extended education^23^. Professional scientists are as accurate as younger adults at verifying statements related to naive ideas, but the lag in response times between naive and matched control statements is larger than for younger adults^24^. Similar findings are observed with other scientific reasoning tasks. Physicists and chemists still hold many naive ideas about thermal phenomena generally believed by middle school students^30^. Both physicists, whose knowledge is in accord with Newtonian principles, and novices exhibit the same common naive idea related to impetus, i.e., that continuation of motion depends on continued action of a force^31^.

﻿Over the past two decades, exploration of the neural substrate of scientific expertise has shed additional light on the difficulty, even for experts, to cope with the coexistence of naive and scientific ideas^32,33^. Most studies have examined experts and highly competent individuals to understand the transition from naive to scientific ideas as experts are believed to have achieved conceptual change^34–37^. The comparison of brain activation patterns between experts and novices point to a correlation between expertise and increased activity in key brain areas of inhibitory control, including the bilateral inferior frontal gyrus (IFG)^38–41^, middle frontal gyrus (MFG)^42–44^, and to a certain extent the anterior cingulate cortex (ACC), ﻿as recent models have focused on ACC’s contribution to effortful control^45^. Masson et al^36^. compared brain activation patterns between ﻿physics undergraduates (at least five college courses) to arts and humanities undergraduates (no high school or college physics courses), when evaluating simple electric circuits diagrams to expose the “single‐wire” naive idea and found increased activity in the IFG, MFG, ACC, as well as in the angular gyrus for physics undergraduates. Using a similar approach, Brault Foisy et al.^34^ showed that undergraduate physics students successfully assessing the correctness of movies with free‐falling bodies (which trigger the “heavier objects fall faster” naive idea) recruited more significantly the prefrontal brain network comprising the IFG and MFG compare to humanities students who can’t overcome their naive ideas about free fall. Allaire-Duquette et al.’s^46^ findings suggest stronger recruitment of the IFG and the MFG in more competent high school students confronted with naive ideas across a wide range of scientific domains. Ultimately, results of all these studies show that the IFG, the MFG and the ACC were significantly more activated in more competent individuals. These findings support the hypothesis of a relation between conceptual change and inhibitory control, i.e., the suppression of prepotent ideas or thoughts to protect working memory’s mental workspace^47^. One possible explanation is that even in experts, naive ideas have not been erased or replaced after learning scientific ideas; they are still encoded in neural circuits that must be inhibited to provide scientifically appropriate responses. However, no neuroimaging study has yet explored how persistent these naive ideas are since the experts examined so far are at most undergraduates. What happens to naive ideas in the brain of scientists with a Ph.D. who have gone through extensive scientific training remains to be explored.

In the present study, we go one step further by examining how naive ideas are processed by scientists with a Ph.D. in physics. We measured brain activity associated with judging the scientific value of statements related to naive ideas in their domain of expertise (physics), or a domain in which they have a more basic level of expertise (biology). We used an experimental task featuring incongruent statements intended to trigger a commonly held naive idea of a given natural phenomenon that leads to a scientifically inappropriate judgement. We also used matched congruent statements triggering the same naive idea, but in a scenario where there is no discrepancy between the naive and scientific ideas therefore the naive idea leads to a scientifically appropriate judgement. Based on previous studies, we hypothesize that evaluating incongruent statements in physics and biology will be associated with increased activity in brain regions, including IFG, MFG, and ACC, while acknowledging that functional specificity related to each domain could vary. Indeed, previous findings with undergraduates suggest that inferior and middle prefrontal brain areas, as well as ACC should be more strongly recruited when scientists with a Ph.D. in physics are confronted with naive ideas (incongruent statements) in biology compared to physics, since no advanced training has targeted them and therefore naive ideas are probably still prevailing strongly^34,36^. Undoubtedly, physicists have been repeatedly exposed to a large number of problems and scientific ideas in physics; therefore, they have less relative exposure to naive ideas as compared to scientific ideas in physics than in biology. On the one hand, this could cause naive ideas in physics to prevail less in physicists^28^. It could also be argued that greater expertise in physics will be related to more efficient inhibition of naive ideas rather than the naive idea being less prevalent. This prediction would be compatible with studies showing that the inhibition of a prepotent response might become easier with age and increased competency^48^. A third alternative explanation could be that the scientific ideas become more prevalent or that physicists even develop a scientific intuition, as suggested by De Neys^49^ or Bago and De Neys^50^. All of three proposed explanations support the prediction that key inhibitory control brain areas (IFG, MFG, and ACC) might potentially be less recruited, when confronted with naive ideas in physics compared to biology. However, it should be noted that our findings don’t allow to test for the different account of these alternative explanations, and that future work could address this finer grain exploration of the relation between expertise and processing of naive ideas.

## Results

### Behavioral data

**Fig. 1:**
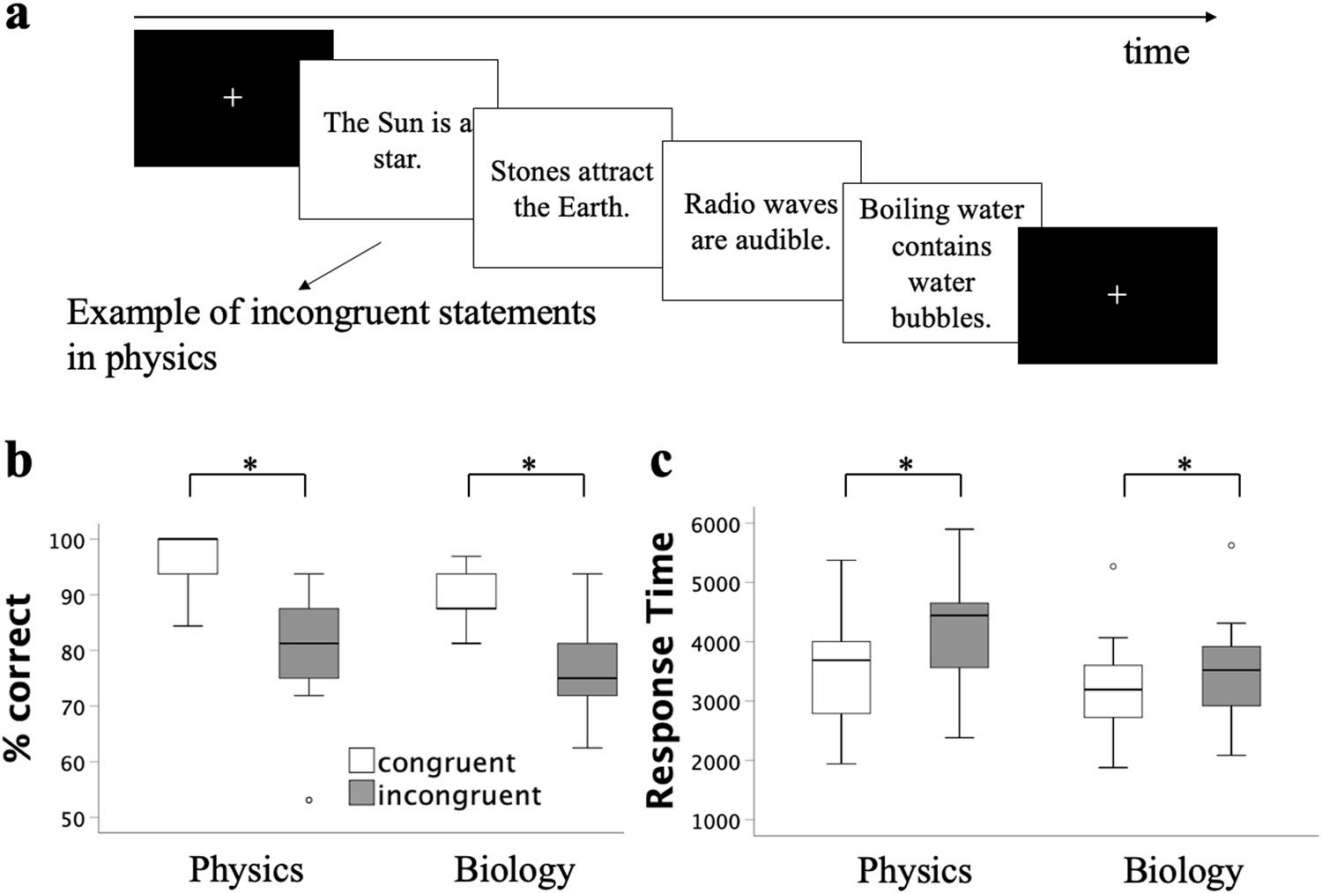
Behavioral results. **a** On each trial, participants were presented with a written statement and judged it as scientifically correct or incorrect. **b** Performance in this task (% correct). **c** Mean response time in this task (ms). Center line represents median; box limits, upper and lower box limits represent quartile 1 and quartile 3; whiskers represent minimum and maximum values; ° represents potential outliers ± 1.5× IQR (interquartile range). *p < 0.001.

As expected, physicists are more accurate in assessing physics statements than biology statements (*t*(24) = 4.360, *p* < 0.001, *d* = 0.5 (*d* = (*M*_1_ − *M*_2_)/*S*_p_, where *M*_1_ and *M*_2_ denote the sample means for groups 1 and 2, and *S*_p_ denotes the pooled estimated population standard deviation), CI 95% [−8.0, −2.9]; *x̄*_physics_ 88.4 ± 11.4% [mean ± standard deviation]; *x̄*_biology_ 83.0 ± 9.0%) although the difference is small (5.4%; Fig. 1b). In physics and biology, participants were more accurate at verifying the scientific value of the congruent statements relative to incongruent ones (Fig. 1b). (Physics: *t*(24) = 8.522, *p* < 0.001, *d* = 1.7, CI 95% [11.8, 19.4]; *x̄*_congruent_ 96.3 ± 4.9%; *x̄*_incongruent_ 80.6 ± 10.6%. Biology: *t*(24) = 8.888, *p* < 0.001, *d* = 1.5, CI 95% [10.2, 16.3]; *x̄*_congruent_ 89.6 ± 4.9%; *x̄*_incongruent_ 76.4 ± 7.1%; chance = 50%). Verifying the scientific value of the congruent statements relative to incongruent ones was also faster in both areas of expertise (Fig. 1c). (Physics: *t*(24) = 10.988, *p* < 0.001, *d* = 2.1, CI 95% [−759.1, −519.0]; *x̄*_congruent_ 3542 ± 754 ms; *x̄*_incongruent_ = 4181 ± 811 ms. Biology: *t*(24) = 4.830, *p* < 0.001, *d* = 0.9, CI 95% [−389.1, −156.2]; *x̄*_congruent_ 3182 ± 699 ms; *x̄*_incongruent_ 3455 ± 749 ms). In physics, response time increased twice as much as in biology between congruent and incongruent statements (Δ_physics_ = +639 ms or 18%; Δ_biology_ = +273 ms or 9%).

### Congruency-dependent modulation of BOLD signal

**Fig. 2:**
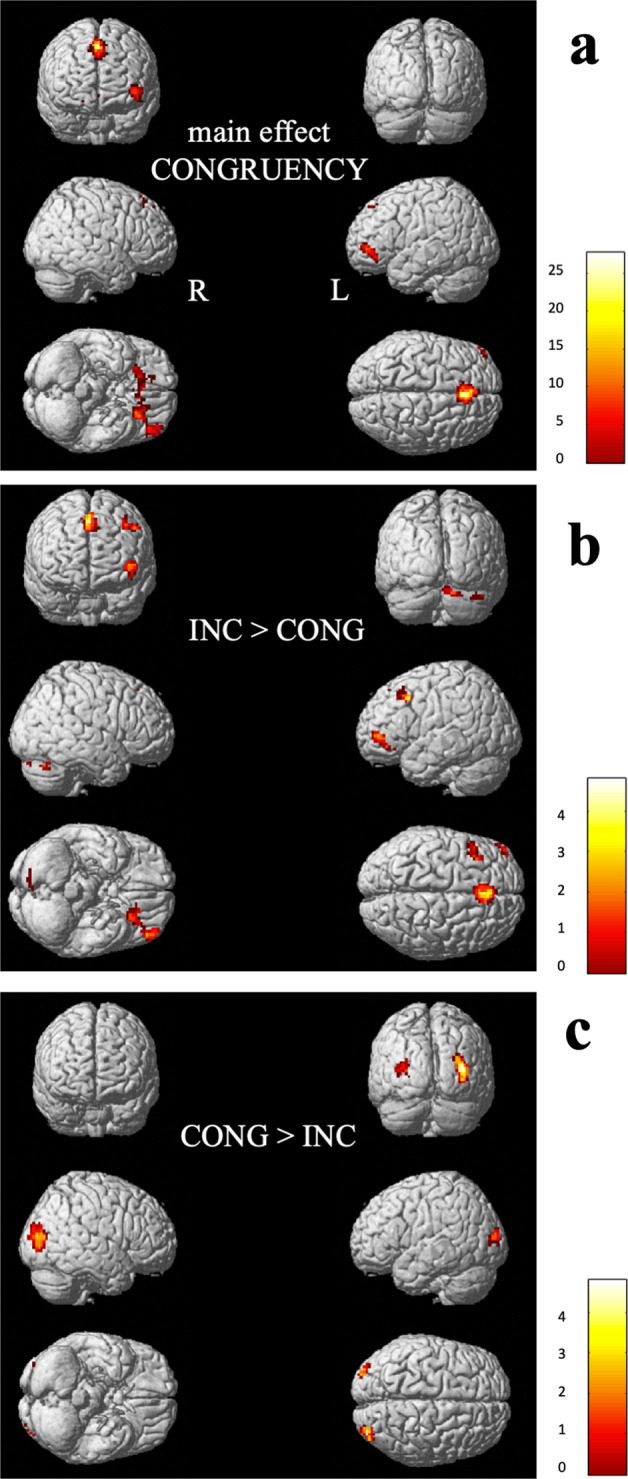
Activation clusters. **a** Main effect CONGRUENCY; subsequent *t*-contrasts between **b** incongruent > congruent statements; **c** congruent statements > incongruent statements. Clusters are presented at *p*_UNCORRECTED_ < 0.005 and are ﻿depicted in the standard single‐subject volume‐rendered brain implemented in SPM8. L left, R right.

We first explored cortical sites where brain responses are modulated by congruency, when expertise is averaged across levels and consequently, which activations are higher for incongruent statements compared to congruent statements or conversely. The results show different brain activation patterns of scientists judging the scientific value of incongruent statements compared to congruent statements (Table 1 and Fig. 2). The analysis identified a set of areas in the left IFG, bilateral superior frontal gyrus, and bilateral ACC. Higher blood oxygen level‐dependent (BOLD) response in all three clusters was found for incongruent statements compared to congruent statements. Additional areas are identified by *t*-contrasts and reveal that incongruent statements, more than congruent ones, activated also the bilateral cerebellum and the MFG, while conversely the congruent statements activated the bilateral middle occipital gyrus more than incongruent ones.

**Table 1:** Significant activation clusters for the main effect CONGRUENCY and the subsequent *t*-contrasts between congruent and incongruent statements.

Brain areas	MNI peak coordinate			Main effect of CONGRUENCY			Incongruent > congruent			Congruent > incongruent		
	*x*	*y*	*z*	*k*	*F*	*p*_FWE-corr_	*k*	*t*	*p*_uncorr_	*k*	*t*	*p*_uncorr_
*Frontal lobe*												
L Inferior frontal gyrus (orbital), R Superior frontal gyrus (orbital), bilateral anterior cingulate gyrus	−21	24	−9	245	27.34	0.002	118	4.90	<0.001	—	—	n. s.
Bilateral superior frontal gyrus (medial)	0	27	51	259	21.49	0.002	180	4.46	<0.0001	—	—	n. s.
L Middle frontal gyrus	−51	15	42	—	—	n. s.	49	3.75	<0.0001	—	—	n. s.
*Occipital lobe*												
R Middle occipital gyrus	39	−84	9	—	—	n. s.	—	—	n. s.	175	4.63	<0.0001
L Middle occipital gyrus	−30	−96	18	—	—	n. s.	—	—	n. s.	49	3.62	0.001
*Hindbrain*												
R Cerebellum crus I	33	−66	−33	—	—	n. s.	39	4.03	<0.0001	—	—	n. s.
R Cerebellum crus II	6	−84	−24	—	—	n. s.	33	3.70	<0.0001	—	—	n. s.

Note: coordinates are reported in MNI space as given by SPM8, main effect at *p*_FWE-CORRECTED_ < 0.05 and *t*-contrasts at *p*_UNCORRECTED_ < 0.005, expected voxels per cluster *k* = 11 for main effect and *k* = 14 for *t*-contrasts, second‐level analysis (random effect analysis, full factorial, and *t* tests). ﻿Coordinates are reported in MNI space as given by SPM8. Anatomical labels are based on the AAL (automated anatomical labeling) atlas (Tzourio-Mazoyer et al., 2002). ﻿The first label represents the location of the peak activation, additional labels denote submaxima if located in a different brain region.

*L* left, *R* right.

### “Expertise × congruency’-dependent modulation of BOLD signal

**Fig. 3:**
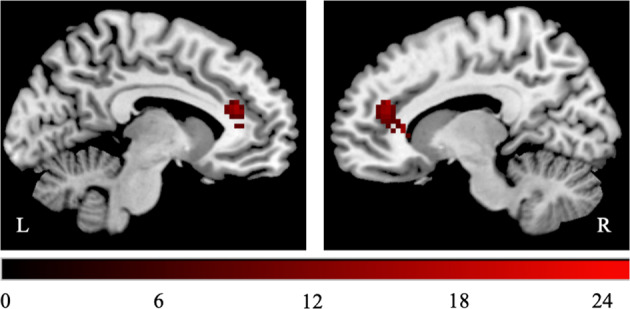
Activation cluster for the interaction effect between EXPERTISE and CONGRUENCY ﻿depicted in the ch2better template in MRIcron107 at pFWE CLUSTER LEVEL < 0.05. L left, R right.

We then searched for differences in activation profiles for the congruent and incongruent statements, depending on the area of expertise (i.e., physics and biology). This interaction effect identified a cluster in the bilateral dorsal ACC (Table 2 and Fig. 3).

**Table 2:** Significant activation cluster for the interaction effect between EXPERTISE and CONGRUENCY.

Brain areas	MNI peak coordinate			EXPERTISE × CONGRUENCY		
	*x*	*Y*	*z*	*k*	*F*	*p*_FWE-corr_
*Frontal lobe*						
Bilateral dorsal anterior cingulate cortex	6	24	0	190	24.35	0.01

Note: coordinates are reported in MNI space as given by SPM8, *p*_FWE CLUSTER LEVEL_ < 0.05, expected voxels per cluster *k* = 11, second‐level analysis (random effect analysis, full factorial), MNI coordinates in mm. Anatomical labels are based on the AAL (automated anatomical labeling) atlas (Tzourio-Mazoyer et al., 2002).

**Fig. 4:**
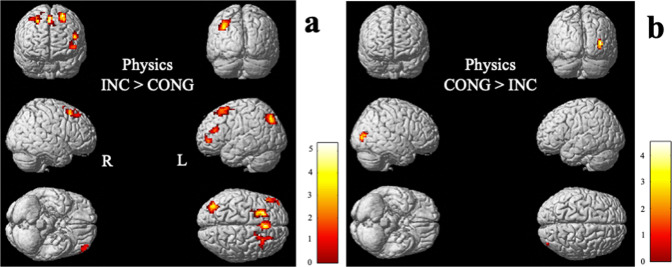
Activation clusters for t-contrasts in physics. **a** Incongruent statements > congruent statement; **b** congruent statements > incongruent statement. Clusters are presented at *p*_UNCORRECTED_ < 0.005 and are ﻿depicted in the standard single‐subject volume‐rendered brain implemented in SPM8. L left, R right.

**Fig. 5:**
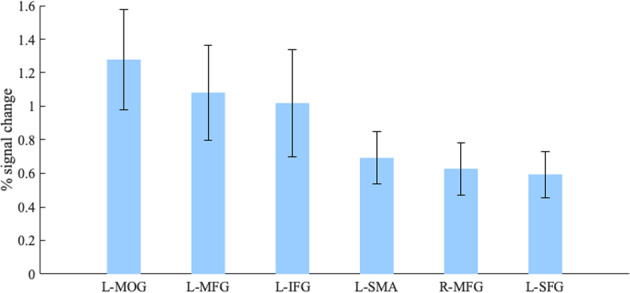
The contrast incongruent > congruent statements in physics displayed with plots of the percentage BOLD signal change from the peak voxels (*p*_UNCORRECTED_ < 0.005; ﻿L left hemisphere, R right hemisphere). Error bars represent SE. L left, R right, MOG middle occipital gyrus, MFG middle frontal gyrus, IFG inferior frontal gyrus, SMA supplementary motor area, SFG superior frontal gyrus. Error bars represent one SEM (standard error of the mean).

Then, we assessed the direction of the interaction effect. We examined which regions were more activated by incongruent or congruent statements for biology and physics independently. In physics (Table 3 and Fig. 4), areas more activated for incongruent than congruent statements include left supplementary motor area, left middle occipital gyrus, left superior frontal gyrus, bilateral MFG, and left IFG. The right middle occipital gyrus is the only area more activated for congruent than incongruent statements. The changes in BOLD signal for each area are presented in Fig. 5.

**Table 3:** Activation clusters for *t*-contrasts between congruent and incongruent statements in physics.

Brain areas	MNI peak coordinate					
	*x*	*y*	*z*	*k*	*t*	*p*_uncorr_
				INCONGRUENT > CONGRUENT		
*Frontal lobe*						
L Supplementary motor area	−3	24	48	158	5.34	<0.0001
L Superior frontal gyrus (dorsolateral)	−21	21	57	169	4.50	<0.0001
R Middle frontal gyrus	33	21	54	127	4.31	<0.0001
L Inferior frontal gyrus (triangular)	−48	33	21	64	4.27	<0.0001
L Middle frontal gyrus	−42	54	3	55	4.20	<0.0001
*Occipital lobe*						
L Middle occipital gyrus	−39	−78	39	153	4.81	<0.0001
				CONGRUENT > INCONGRUENT		
*Occipital lobe*						
R Middle occipital gyrus	39	−84	9	63	3.95	<0.0001

Note: coordinates are reported in MNI space as given by SPM8, *p*_UNCORRECTED_ < 0.005, expected voxels per cluster *k* = 11, second‐level analysis (random effect analysis, *t* tests), MNI coordinates in mm. Anatomical labels are based on the AAL (automated anatomical labeling) atlas (Tzourio-Mazoyer et al., 2002).

*L* left, *R* right.

**Fig. 6:**
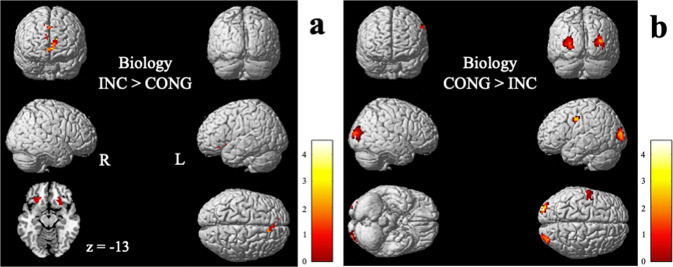
Activation clusters for *t*-contrasts associated with statements in biology. **a** Incongruent statements > congruent statement; **b** congruent statements > incongruent statement. Clusters are presented at *p*_UNCORRECTED_ < 0.005 and 3D renders are ﻿depicted in the standard single‐subject volume‐rendered brain implemented in SPM8, while 2D renders are presented in the ch2better template in MRIcron^107^. L left, R right.

**Fig. 7:**
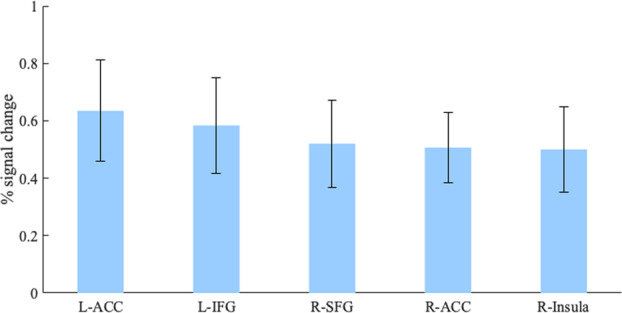
The contrast incongruent > congruent statements in biology displayed with plots of the percentage BOLD signal change from the peak voxels (*p*_UNCORRECTED_ 0.005; L left hemisphere, R right hemisphere). Error bars represent SE. L left, R right, ACC anterior cingulate cortex, IFG inferior frontal gyrus, SFG superior frontal gyrus. Error bars represent one SEM (standard error of the mean).

In regard to statements in biology (Table 4 and Fig. 6), areas more activated for incongruent than congruent statements include left IFG, bilateral ACC, bilateral caudate, right insular cortex, bilateral superior frontal gyrus, and right midcingulate gyrus. Areas more activated for congruent than incongruent statements are comprised of the bilateral middle occipital gyrus and left precentral gyrus. *T*-contrasts reveal that the interaction effect is attributed to the activation pattern of processing of incongruent statements in biology for which the participants possess basic expertise. The changes in BOLD signal for each area are presented in Fig. 7.

**Table 4:** Activation clusters for *t*-contrasts between congruent and incongruent statements in biology.

Brain areas	MNI peak coordinate					
	*x*	*Y*	*z*	*k*	*t*	*p*_uncorr_
				INCONGRUENT > CONGRUENT		
*Frontal lobe*						
L Inferior frontal gyrus (triangular), Bilateral caudate	−21	30	−12	83	4.53	<0.0001
R Anterior cingulate cortex	12	39	24	40	3.97	<0.0001
R Insula, R Superior frontal gyrus (orbital)	27	21	−15	30	3.95	<0.0001
L Anterior cingulate gyrus, R Superior frontal gyrus (medial orbital)	−15	33	21	105	3.86	<0.0001
R Superior frontal gyrus (medial), R Midcingulate gyrus	9	33	39	34	3.72	<0.001
				CONGRUENT > INCONGRUENT		
*Frontal lobe*						
L Precentral gyrus	−48	−6	42	62	3.87	<0.0001
*Occipital lobe*						
R Middle occipital gyrus	33	−93	12	120	4.67	<0.0001
L Middle occipital gyrus	−27	−96	9	128	4.36	<0.0001

Note: coordinates are reported in MNI space as given by SPM8, *p*_UNCORRECTED_ < 0.005, expected voxels per cluster *k* = 12, second‐level analysis (random effect analysis, *t* tests), MNI coordinates in mm. Anatomical labels are based on the AAL (automated anatomical labeling) atlas (Tzourio-Mazoyer et al., 2002). The first label represents the location of the peak activation, additional labels denote submaxima if located in a different brain region.

*L* left, *R* right.

## Discussion

﻿In this functional magnetic resonance image (fMRI) study, congruent and incongruent statements about natural phenomena were presented to scientists with a Ph.D. in physics. Each incongruent statement relates to a naive idea that interferes with a scientifically appropriate judgment, while otherwise equivalent congruent statements triggered the same naive idea, but in a scenario where there is no discrepancy between the naive and scientific ideas. We searched for greater activations to incongruent than to congruent statements to investigate what happens in experts’ mind when confronted with commonly held naive ideas that interfere with a scientifically valid judgment they have been trained to perform. Our hypothesis was twofold. First, activity in IFG, MFG, and ACC will be greater for naive ideas compared to control ones. Naive ideas are thought to be deeply rooted in humans’ cognition and are often reinforced by everyday life experiences. They are often spontaneous, accompanied by a feeling of certainty^28,51^, and are subject to “recency effect”^52^. This hypothesis is based on findings suggesting that even advanced undergraduate students in physics may still hold to naive ideas of science encoded in neural networks that must be inhibited in order to answer scientifically^34,36^. Secondly, we also hypothesized that the activation of inhibitory control is likely more prominent for statements in biology since participants possess a more basic level of expertise in this area compared to physics. Indeed, despite being persistent, the prevalence of naive ideas appears to decrease with age/expertise. Potvin and Cyr^28^ found that science teachers’ responses to a buoyancy task are faster and more accurate than preschoolers’, elementary students’, and secondary students’ responses. Lanoë, Vidal, Lubin, Houdé, and Borst^53^ observed that experts in mathematics need to inhibit naive ideas when solving arithmetic word problems, but the negative priming effect (effect of prior exposure to naive ideas on subsequent responses) was of smaller amplitude than in nonexperts.

Performance data show that incongruent statements displayed lower accuracy and longer response time than congruent statements, reflecting the interference and performance decrement caused by the contextually irrelevant information^54^ of naive ideas. These findings, observed for physics as well as for biology statements, suggest that even with their expertise, physicists’ scientific judgment is still impacted by interfering naive ideas in science. ﻿Indeed, when asked to evaluate the scientific value of the statements, participants were more likely to rely on naive ideas of given phenomena, as they are still easily accessible and often reinforced in real‐life contexts^21^. This effect has been repeatedly found in studies on interference and inhibitory control mechanisms for different age groups﻿^28,55^ and our results are in line with previous findings demonstrating that participants, including experts, verify statements whose truth value differs from naive ideas (incongruent situations) more slowly and less accurately than statements, where naive ideas do not interfere^23–25,29–31^﻿.

Interestingly, as did Shtulman and Legare^56^﻿, we found a larger response lag between congruent and incongruent statements in physics than in biology. However, unlike Shtulman and Legare^56^﻿ it is unlikely attributable to our participants being less knowledgeable about matter than life sciences. It is more likely that participants’ advanced expertise in physics was associated with increased conceptual reasoning. The comparison between the two domains remains speculative as statements were not matched for word and syllable counts between domains. In sum, overcoming the interference of naive ideas appears to require increased inhibitory control, and therefore might explain longer response times in incongruent compared to congruent trials, as previously shown^23,57^﻿.

Physicists yielded broad extent of activation when confronted with incongruent statements (Fig. 4a), while they revealed a smaller and more focal activation when confronted with congruent statements (Fig. 4b). Moreover, the spatial distinction was mainly observed in frontal regions for incongruent and in a posterior area for congruent statements. The greater involvement of the frontal and prefrontal regions is interpreted as evidence of increased attentional demand. One plausible explanation is enhanced recruitment of cognitive control processes for suppressing a dominant response, the naive idea, and selecting a relevant but less salient response. Two brain areas, the MFG and IFG, associated with inhibitory control mechanisms were significantly more activated by incongruent statements. The MFG is generally associated with executive control processes including inhibitory control, working memory and task switching^58^. This region is found to be particularly active when blocking prepotent responses^44^, as well as reorienting to unexpected stimuli^59,60^. Neuroimaging studies have also highlighted how the IFG shows increased activation when suppressing prepotent responses^38–41,59,60^, and findings suggest that that the integrity of left IFG is also critical for the successful implementation of inhibitory control^61^. Moreover, although multiple studies have also associated inhibitory control with the right IFG^39,62,63^, the left IFG is more likely activated by semantic inhibitory control^64–67^, which concurs with the verbal nature of the misconception-oriented task used in our study.

These findings are in line with our hypothesis and provide evidence that even experts with a Ph.D. would need to suppress naive ideas in science to reason scientifically. Naive ideas are indeed known to be more easily accessible and are reinforced in everyday situations^21^. They plausibly rely on neural pathways with increased synaptic efficacy arising from repeated and persistent recruitment^68^. Findings are also in close agreement with previous findings of ﻿stronger recruitment of IFG, MFG, and ACC for successfully overcoming naive ideas in science^34,36,46,69^. Contrary to our hypothesis, the ACC didn’t show increased activity for incongruent compared to congruent statements in physics. In our study, the BOLD response in the ACC is modulated by the incongruency of statements in biology (Fig. 6a) which will be the topic of the next subsection.

Resisting naive ideas in physics also recruited more strongly the supplementary motor area that is usually associated with organizing and preparing voluntary movement, that is, the readiness for action^70^. The left supplementary motor area would also play a more important role in controlling hand movements in right-handed individuals^71^ (in this study, our right-handed participants needed to press a button with the right hand). The anterior part of the supplementary motor area (the pre-SMA) is well known to by associated to inhibitory control^39,72^, and is structurally connected to the IFG^73^, a key region in inhibitory control. Moreover, increased demand on working memory and identification of meaning could have been more critical for reading incongruent statements, as evidenced by the higher activity found in the superior frontal gyrus and middle occipital gyrus. The first region is thought to particularly contribute to working memory^74^. The second region is implicated in visual word form processing^75^ that includes the identification of shapes, letters, and words prior to, or in parallel to, identification of sound and/or meaning^76^.

Congruent statements, for their part, showed increased recruitment of a foci in the right middle occipital gyrus. This region has been found to be involved in a diversity of networks, including spatial and semantic processing^77,78^, but not in networks related to executive and inhibitory control. Therefore, our data provides evidence for a more automatic/perceptual processing of congruent statements by physicists, which is coherent with view of neural efficiency as a basis for optimal cognitive expertise with less and confined activations. Automatic processing of experts is associated with more selective brain areas that only manage the respective task demands in parallel with attenuating the dependency on controlled processes from the frontal areas^79^.

Overall, the brain activation patterns associated with processing naive ideas in physics seem to overlap with the nodes of the dorsal attention system in addition to the left IFG that might reflect more flexible attentional resources^80^. While the dorsal network is known to be specialized in top-down controlled attentional selection, the left IFG’s activity can be correlated to response suppression and reorientation of attention to relevant information that it important, but not obvious^81^. Hence, experts in this study are likely still burdened by the interference of naive ideas that are successfully controlled and inhibited. Since the task involved different notions, these findings appear to hold true for multiple concepts in physics: gravity, matter, heat, etc.

Comparably to physics, in biology, participants revealed a broader range of activations during the assessment of incongruent statements, whereas they revealed more focal activations for congruent statements. Our data revealed five brain regions that mediate the performance of physicists with a Ph.D. assessing naive science ideas in biology. As mentioned above, the IFG shows increased activation when suppressing prepotent responses^38–41^, and findings suggest that that the recruitment of left IFG is also critical for the successful implementation of inhibitory control^61^. The ACC is implicated with error detection and decision-making^82^. It detects that a particular situation or task requires higher cognitive control. Consequently, the ACC may be the brain region that triggers the inhibition process. Fugelsang and Dunbar^32^ found increased activity in the anterior cingulate when medical students evaluate data inconsistent with a plausible theory on the effectiveness of a drug. The ﻿right insula has been implicated consistently in inhibitory control and shows stronger functional connectivity with the ACC^62^. Interestingly, our experts had a noticeable error rate (17%) when evaluating statements in biology and the right insula specifically shows greater activation during unsuccessful trials of response inhibition tasks^62^. Similarly to the right insula, the median cingulate gyrus is more activated by failed compared to successful inhibition^83^. The right superior frontal gyrus plays a role in the control of impulsive response, suggesting its function in action restraint^84^. In other words, during conflict anticipation the right superior frontal gyrus is positively correlated with the capacity of inhibitory control.

Congruent statements, for their part, showed increased recruitment of a foci in the bilateral middle occipital gyrus. This brain area has been found to be involved in a diversity of networks, including spatial and semantic processing^77,78^, but not in networks related to executive and inhibitory control. The foci in the left precentral gyrus is the site for primary motor cortex. Although the predominant role of this region is motor execution, more recent studies propose that it also plays a key role in higher cognitive processes, such as attention and movement inhibition^85^. The left primary motor cortex potentially contributed to controlling hand movements in our right-handed participants who use their right hand to push the response box button. In sum, similarly to the case of physics, our data suggests a more automatic/perceptual processing of congruent statements in biology.

The brain activation patterns associated with processing naive ideas in biology seem to largely overlap with the nodes of inhibitory control mechanisms. The IFG, the ACC, the insula, and the superior frontal gyrus have repeatedly been found to be involved in inhibitory control mechanisms in a wide range of cognitive tasks. Conversely, our data also provides evidence for a more automatic/perceptual processing of congruent statements in biology. Taken together, these results suggest that physicists are likely still burdened by the interference of naive ideas in biology. To respond successfully to incongruent statements, they must control and inhibit common naive ideas. Like in physics, the task involved different notions, therefore these findings appear to hold true for a diversity of biology concepts.

One of the findings worth noting in the interaction analysis is that being confronted with naive ideas for which one possesses a basic versus an advanced level of expertise reveals rather separable activation patterns, but with notable overlapping activity in the IFG, pointing toward a central role for this brain area in processing interferences, including naive ideas in science, even for individuals having an advanced expertise. Indeed, the literature on IFG function specifically motivates its critical involvement in inhibitory control. Behavioral performance also supports the implication of inhibition as accuracy was significantly lower and response time slower for incongruent statements compared to congruent ones for the two levels of expertise. Yet, direct comparisons between biology and physics should be interpreted with caution as it could be allocated to other factors, including differences in the scientific content of the statements in the two areas. Furthermore, there are some inherent epistemological distinctions between physics and biology that could also cause the differences observed between biology and physics statements. Hence, it is unclear to what extent the brain activations comparing specific brain networks related to biology vs. physics are due to the level of expertise vs. the nature of the disciplines themselves and possibly other confounding factors including the respective curricula of the two disciplines. In addition, the time elapsed between physics and biology studies and the experiment differ greatly in our sample of physics experts, thus adding a confounding variable to the comparison of the two disciplines. Therefore, results regarding the expertise-dependent modulation of brain activity are not addressed directly in this study but are made available in Supplementary Material (see Supplementary Discussion—Main effect of expertise). Future study should address these limitations to better understand the inherent differences in brain-based mechanisms between advanced vs. basic expertise.

Although resisting to naive ideas in biology and physics reveal overlapping brain activations associated with inhibitory control, there is one significant difference. As evidence by the interaction effect between expertise and congruence, the main difference between physicists confronting naive ideas in physics and biology lies in the activity of the ACC. The direction of the interaction effect is driven by the contrast “incongruent > congruent statements” showing a higher BOLD response during the assessment of biology statements. ﻿As previously mentioned, the ACC is well known for its role in error detection and conflict monitoring^82^. According to this view, it appears plausible that the correct assessment of incongruent statements in both biology and physics requires inhibition. However, the certainty of the responses for incongruent statements in biology is lower since physicists are not advanced experts in biology and therefore, they are likely to have automated the processing of naive ideas in biology more than in physics due to lower exposure to scientific ideas in biology. The uncertainty might cause a stronger activation in the AAC. This interpretation is compatible with the literature on decision-making under uncertainty showing that when we have doubts about our responses, the ACC is more activated^51^. The recruitment of the IFG without a significant contribution of the ACC has been observed in situations about free falling objects in which inhibitory control is required, but in which there is no doubt about the correctness of the responses^34^. In sum, the main difference between physicists resisting to naive ideas in physics and biology doesn’t appear to be the involvement of inhibition, but the certainty of the provided responses.

It should be noted that one of the main limitations of this study is that the results are drawn from a reverse inference, where patterns of brain activity are used to infer the engagement of mental processes, such as inhibitory control, working memory, etc.^86^. Contrary to a forward inference where, based on a given psychological manipulation, one can infer correlated changes in brain activity, reverse inference can be more problematic because several mental processes share similar neural patterns^87^. Thus, when demonstrating the engagement of a mental process from a pattern of brain activity like it is done in this study, one should remember that the interpretation is still a hypothesis that would need to pass a rather higher standard of proof to be likely considered a conclusive finding^88^. Nonetheless, although we share the view that reckless use of reverse inference is highly problematic, we also argue that reverse inference can have a predictive power and its reliability is dependent notably on the design of the cognitive task used as demonstrated in the revised formulation of reverse inference^89^. Conditioning by task as we do here increases the predictive power of the presence of a specific cognitive process. For instance, in the case of this study, the experimental context didn’t focus on all activations associated with processing naive ideas in science, but rather activations associated with processing incongruent ideas in science more than congruent ideas in science. Nonetheless, we prudently recommended that these findings be treated with considerable caution.

To evaluate how persistent some naive ideas about natural phenomena are, we measured BOLD signals of 25 scientists with a Ph.D. in physics assessing the scientific value of incongruent and congruent statements, where naive ideas interfere with an appropriate scientific judgement or not, related to their domain of expertise (physics) and a domain, in which they have a more basic level of expertise (biology). In accordance to our hypothesis, the overall extent of activations for assessing incongruent statements was larger than for assessing congruent statements, especially in frontal areas, including IFG and MFG. Since these two brain regions are known to be involved in inhibitory control, and since the activations are observed for incongruent but not for congruent statements, these results suggest that inhibitory control could play a role when scientists with a Ph.D. in physics assess naive ideas in their field of expertise and in biology, where they have a more basic level of expertise.

Our findings might seem surprising since expertise is usually associated with neural efficiency, meaning less and confined activations. Through practice, processing becomes automatic requiring less effort and fewer procedural steps^90–92^. However, while expertise is generally reflected by smaller and more selective brain areas^79^, it is likely that even advanced experts have difficulty preserving cognitive resources when being confronted with deeply rooted naive science ideas. Indeed, they are likely to require increased active control to block unwanted thoughts that stem from naive ideas. ﻿This finding is in strong agreement with previous evidence from behavioral studies on the interference of naive ideas in scientific reasoning^23,24,28,93^, and neuroscientific studies on scientific expertise^34,36,46^. Taken together, these results suggest that some naive ideas are extremely persistent, and probably never disappear, even after an extensive training, such as a Ph.D. in physics. Indeed, while certain early acquired ideas or stories appear to be more ephemeral, such as when children learn to differentiate between ordinary and magical events (e.g., moving a marble with one’s mind)^94^, many naive ideas are different from those generally accepted by the scientific community and have proven to persist despite formal scientific instruction. They may persist because they are based on deeply anchored intuitions or heuristics^14,25,28^ or because they are more reinforced in everyday life contexts compared to scientific conceptions^2–4,20,21^.

A finer grain analysis of the differences between processing naive ideas in biology and physics by scientists with a Ph.D. reveals that, while both seem to increase the activity of IFG and MFG, biology incongruent statements were associated with a specific increased activity in the ACC. Since this region can be associated with error detection, conflict motoring, and decision-making under uncertainty, this activation could be related to the fact that, although physicists could successfully inhibit naive ideas in biology, they were not so sure about their answers in biology compared to physics. Therefore, it could be argued that error detection and the ACC plays a more significant role in basic expertise, but that, the necessity for conflict monitoring eventually decreases since naive ideas probably don’t prevail as much^28^.

Altogether, our data reveals that a widely distributed network of frontal brain regions associated with executive functions and inhibitory control mediate the performance of experts, when judging the scientific value of statements related to naive science, whereas matched statements that do not relate to naive science hinge on a more selected and focal posterior brain regions associated with automatic processing of information. These results evidence a perspective according to which naive ideas of science are unlikely to be replaced by scientific ones and that, in the long run, conceptual change as it was initially described (a replacement of naive ideas with scientific ones) might never truly be achieved. Conceptual change might instead yield multiple ideas of each given natural phenomena, including scientific and naive ideas, which then have to be coordinated to demonstrate appropriate conceptual understanding. Instead, results encourage us to reconsider scientific expertise, suggesting that experts’ outstanding performance and success may be less an attainment and more a dynamic vigilance, allowing active control of thoughts when the context requires for it. In sum, this study is a springboard that lead to envision a larger scale study of expertise and conceptual change in sciences in different scientific fields. Future studies should also track changes in behavioral and brain activity, as novice participants gradually acquire expertise and experience conceptual change over time.

## Methods

### Participants

We scanned a total of 25 right-handed adults who complete a Ph.D. in physics, including 23 male and 2 female participants (age range 28–60 years, *x̄* = 45 years). The sample comprised university professors (*n* = 17), postdoctoral researchers (*n* = 3), senior high school (year 12 and 13), teachers (*n* = 5), and a developer–researcher (*n* = 1). All participants gave written informed consent and were duly compensated for their participation. None of the participants reported any abnormal neurological history. All experimental procedures were approved by the Research Ethics Board of Quebec’s Neuroimaging Network (CMER RNQ 13-14-023) endorsed by the Ministry of Health and Social Services of Quebec (Canada). We would like to acknowledge that there is a need for more studies to incorporate sex as a variable in experimental designs^95^. The incapacity to explore these differences due to small sample size represents a limitation of this study, but can be largely explained by contextual factors beyond our control. Women remain underrepresented in physics. Although we have seen many improvements in the past decades, the percentage of women faculty in physics is, unfortunately, still stagnant at 12%^96^.

### Behavioral task

Participants were presented with naive ideas in a scientific statements task (see Supplementary Material—Supplementary Methods). On each trial, a short-written sentence was displayed for a 10-s period maximum during which the participants had to indicate whether the statement was scientifically correct or incorrect by pressing a button (Fig. 1a). Instructions stressed both speed and accuracy. They were given orally by the experimenter and then again visually on the computer screen before starting the task. The task includes 128 scientific statements: 64 statements address phenomena in physics and 64 statements address phenomena in biology. Statements in the two scientific fields are equivalent for readability (Flesch index_Physics_ = 75.7; Flesch index_Biology_ = 70.2) as indexes for both domains are interpreted as “fairly easy to read —score 70.0–80.0”, corresponding to an average grade seven student’s written assignment^97,98^. The statements in both domains are organized in pairs of incongruent and congruent statements matched on scientific content (concept) and level of analysis (macroscopic or microscopic). Congruent and incongruent statements were also equivalent in terms of response type (64 true and 64 false statements), word count (congruent statements = 566 words, incongruent statements = 568 words; *x̄*_congruent statements_ = 8.8 ± 3.7 [mean ± standard deviation]; *x̄*_incongruent statements_ = 8.9 ± 3.8; *t*(126) = −0.05, *p* = 0.96, 95% CI [−1.35, 1.29]); syllable count (congruent statements = 833 syllables, incongruent statements = 832 syllables; *x̄*_congruent statements_ = 13.0 ± 5.2; *x̄*_incongruent statements_ = 13.0 ± 5.1; *t*(126) = 0.02, *p* = 0.99, 95% CI [−1.79, 1.82]), and readability (Flesch index_congruent statements_ = 72.7, Flesch index_incongruent statements_ = 73.1)^97,98^.

Congruent statements portray situations where naive and scientific ideas are compatible, therefore usually generating higher accuracy. Incongruent statements portray situations where naive and scientific ideas are incompatible, thus where the inherent challenge is to resist the prevailing erroneous naive ideas. For instance, in the congruent statement “a campfire contains thermal energy”, both the naive idea “only hot objects contain thermal energy”^99^, and the scientific idea “thermal energy is the result of the movement of atoms” lead to a scientifically appropriate judgment. However, in the matched incongruent statement “an ice cube contains thermal energy”, the naive idea leads to answer inappropriately that the statement is incorrect, although it is correct according to the scientific idea of thermal energy. The statements were validated with the help of professional physicists and biologists, and addressed naive ideas that are known to be especially persistent, for instance, in mechanics, thermodynamics, natural selection, and photosynthesis.

### Experimental design

The experiment was presented in a block design divided into four runs of eight blocks, comprising four statements each and presented in a random sequence. The trials were presented until the participant responded, or for a maximum duration of 10 s. A fixation cross was displayed for 15 s between each block. In addition, two mixed blocks were randomly added in each run to prevent habituation and included both congruent and incongruent statements. These filler statements were not included in the analysis as they were not matched between congruent and incongruent conditions. The total experiment time was ~20 min. Before the MRI session, a practice task comprising different statements was performed on a desktop computer and in a mock scanner to allow habituation in an environment less daunting than a real scanner. During the practice, participants were presented with 16 trials so that they become familiar with the visual aspect of the stimuli, the sequence of trials, and the response box. No participant asked for additional instructions nor showed signs of confusion when carrying out the practice or when encouraged to ask for clarification.

Stimuli presentation was accomplished with E‐Prime 2.0 software (Psychology Software Tools, inc.) on a projection screen in the scanner room, visible to the participant through a mirror mounted above the head coil. Participants’ responses were collected with a Fiber Optic Button Response System (Psychological Software Tools, inc., Sharpsburg, Pennsylvania, USA). A response box was placed in the participants’ right hand. Participants were instructed to press the response box button with the index finger if they judged the stimuli as “correct” (scientifically appropriate), and they were instructed to press with the middle finger if they judged the stimuli to be “incorrect” (scientifically inappropriate).

### fMRI data acquisition

We used a 3-Tesla (Siemens Prisma) with a 32-channel head coil. To minimize head movement, subjects’ heads were stabilized with foam cushions. Functional images were obtained with a single‐shot gradient echo EPI sequence sensitive to BOLD contrast (TR = 2000 ms, TE = 30 ms, FA = 90°, FOV = 192 mm, matrix size = 64 × 64, pixel size = 3 × 3 mm, interleaved). Thirty‐three 3‐mm‐thick transverse slices with a distance factor of 25% were acquired parallel to the AC–PC line. For each run, between 120 and 200 functional volumes were obtained. The first two volumes were discarded to account for T1 saturation effects. Structural images were obtained using a T1‐weighted 3D MPRAGE sequence (TR = 2,300 ms, TI = 900 ms, TE = 2.26 ms, FA = 9°, FOV = 256 mm, matrix size = 256 × 256, 1 slab, 176 images per slab, pixel spacing = 1 × 1 mm). The acquisition of structural images lasted ~10 min.

### Image processing and analysis

Analysis of structural and functional imaging of the fMRI data was performed using SPM8 software v6313^100^. The functional data of each participant were motion‐corrected, co‐registered with structural data, and then spatially normalized into the standard MNI space (Montreal Neurological Institute). Head motion was corrected with SPM8 across and within sessions of every individual subject. The fMRI series were corrected for motion using the standard SPM “spm_realign” procedure and mean images were calculated. Images are realigned to the mean image. Normalization was performed using the standard SPM “spm_coreg” procedure that co‐registers the individuals T1 to their EPI and allows for a better normalization to the MNI template. The “single generative model” segmentation approach was employed to determine normalization parameters and used to normalize spatially the realigned images to the MNI reference space. This model involves alternates among classification, bias correction, and registration steps. The normalized EPI images were smoothed with a Gaussian kernel of 8 mm FWHM.

A two-level analysis was then implemented. For each participant, fMRI images were high-pass filtered at 128 s to remove low‐frequency drifts. Then, the statistical analysis was conducted using the general linear model. Model time courses for each experimental condition block were generated on the basis of the hemodynamic response function implemented in SPM8. The analysis for the entire group was performed by computing linear *t*‐contrasts (experimental conditions vs. fixation period) for each subject individually, which were then entered into random effects (second-level) whole-brain analysis of variance (ANOVA) using full factorial design analysis in SPM8. Described as a within-within‐subject ANOVA with two within-subject factors of two levels each: EXPERTISE (basic vs. advanced) × CONGRUENCY (congruent vs. incongruent)^101^. Full factorial design is SPM terminology and refers to an option in the interface to specify the design matrix (and non‐sphericity assumptions) of a GLM. The full factorial design analysis was used to model the main effect of CONGRUENCY and the interaction between EXPERTISE × CONGRUENCY to highlight differences in activation patterns between within-subject factors^101^. All brain activations resulting from the ANOVA are reported at the familywise error (FWE)-corrected threshold *p*_FWE-CORRECTED CLUSTER-WISE_ < 0.05 across the whole brain, using a primary voxelwise threshold of *p*_UNCORRECTED_ < 0.005 (in addition, a region of interest analysis can be found in Supplementary Material—Supplementary Discussion). Cluster-extent based thresholding can offer increased sensitivity to detect activations with large spatial extent to studies with moderate sample size^102^. Despite some limitations, cluster‐extent based thresholding has relatively high sensitivity^103,104^ and takes into consideration the fact that voxel activations are not independent of the activations of neighboring voxels, especially when working with spatially smoothed data^105,106^. Post hoc *t-*contrasts revealing the direction of the main effect and interaction (ANOVA) are reported using voxelwise threshold *p*_UNCORRECTED_ < 0.005 to account for their lower statistical power compared to ANOVAs.

We used a primary voxelwise threshold of *p*_UNCORRECTED_ < 0.005. Although Woo et al.^102^ recommend, in general, using more stringent cluster‐defining primary thresholds (*p*_UNCORRECTED_ < 0.001 for a range of typical cases) to reduce the possibility of obtaining false positive clusters and to improve the degree of confidence in inferences about spatial specificity. Our choice is supported by the fact that Woo et al.’s^102^ simulation was based on a sample of *N* = 33, while in this study the sample is *N* = 25, and that our hypothesis regarding the increased activity in IFG, MFG, and ACC areas does not require high spatial specificity like it is the case when studying smaller brain anatomical structures. However, although it appears reasonable to use a more liberal primary voxelwise threshold for this study, findings should be interpreted with some limitations in mind. Indeed, a more liberal cluster-defining primary threshold is associated with larger clusters spanning several anatomical structures^102^. Therefore, one can only suppose that the activation is located within a significant cluster, but one cannot be confident about the statistical significance of specific locations within the cluster^102^. In sum, one can’t reliably infer which brain regions show true effects within the significant cluster.

### Reporting summary

Further information on research design is available in the Nature Research Reporting Summary linked to this article.

## Supplementary information

Reporting Summary

Supplementary Information

## Data Availability

The data that support the findings of this study are available on request from the corresponding authors (S.M. and G.A.D.). The data are not publicly available due the current legal provisions of Quebec (Canada) and in particular the Act respecting the protection of personal information in the private sector (L.R.C. (1985), ch. P-21, http://www.legisquebec.gouv.qc.ca/en/showdoc/cs/P-39.1).

## Code availability

The MATLAB script used for fMRI data preprocessing and statistical analysis is available from the corresponding author, upon reasonable request for purposes of reproducing or extending the analysis.

## References

[1] Vosniadou S (2019). The development of students’ understanding of science. Front. Educ.

[2] Amin, T., Smith, C. & Wiser, M. In *Handbook of Research on Science Education*, (eds. Lederman, N. G. & Abell, S. K.) Vol. II, 57–81 (Routledge, 2014).

[3] diSessa, A. A. in *Cambridge Handbook of the Learning Sciences*, (ed. Sawyer, K. R.) 265–281 (Cambridge University Press, 2006).

[4] Driver R, Easley J (1978). Pupils and paradigms: a review of literature related to concept development in adolescent science students. Stud. Sci. Educ..

[5] Halloun IA, Hestenes D (1985). Common sense concepts about motion. Am. J. Phys..

[6] Baxter J (1989). Children’s understanding of familiar astronomical events. Int. J. Sci. Educ..

[7] Sharp JG (1996). Children’s astronomical beliefs: a preliminary study of year 6 children in South-West England. Int. J. Sci. Educ..

[8] Duit R, Treagust DF (2003). Conceptual change: a powerful framework for improving science teaching and learning. Int. J. Sci. Educ..

[9] Potvin P, Sauriol E, Riopel M (2015). Experimental evidence of the superiority of the prevalence model of conceptual change over the classical models and repetition. J. Res. Sci. Teach..

[10] Kummer TA, Whipple CJ, Jensen JL (2016). Prevalence and persistence of misconceptions in tree thinking. J. Microbiol. Biol. Educ..

[11] Prince M, Vigeant M, Nottis K (2012). Development of the heat and energy concept inventory: preliminary results on the prevalence and persistence of engineering students’ misconceptions. J. Eng. Educ..

[12] Driver R (1989). Students’ conceptions and the learning of science. Int. J. Sci. Educ..

[13] Eylon BS, Linn MC (1988). Learning and instruction: an examination of four research perspectives in science education. Rev. Educ. Res..

[14] De Neys, W. & Goel, V. In *Neuroscience of Decision Making*, (eds. Vartanian, O. & Mandel, D. R.) 125–142 (Psychology Press, 2011).

[15] Chi, M. T. H. in *Cognitive Models of Science*, (eds. Giere, R. & Feigl, H.)129–186 (University of Minnesota Press, 1992).

[16] Galili I, Bar V (1992). Motion implies force: where to expect vestiges of the misconception?. Int. J. Sci. Educ..

[17] Linder CJ (1993). A challenge to conceptual change. Sci. Educ..

[18] Mortimer EF (1995). Conceptual change or conceptual profile change?. Sci. Educ..

[19] Tyson LM, Venville GJ, Harrison AG, Treagust DF (1997). Multidimensional framework for interpreting conceptual change events in the classroom. Sci. Educ..

[20] Ohlsson S (2009). Resubsumption: a possible mechanism for conceptual change and belief revision. Educ. Psychol..

[21] Solomon J (1983). Learning about energy: how pupils think in two domains. Eur. J. Sci. Educ..

[22] Kelemen D, Rosset E (2009). The human function compunction: teleological explanation in adults. Cognition.

[23] Kelemen D, Rottman J, Seston R (2013). Professional physical scientists display tenacious teleological tendencies: purpose-based reasoning as a cognitive default. J. Exp. Psychol..

[24] Shtulman A, Harrington K (2016). Tensions between science and intuition across the lifespan. Top. Cogn. Sci..

[25] Shtulman A, Valcarcel J (2012). Scientific knowledge suppresses but does not supplant earlier intuitions. Cognition.

[26] Brookman-Byrne A, Mareschal D, Tolmie AK, Dumontheil I (2018). Inhibitory control and counterintuitive science and maths reasoning in adolescence. PLoS ONE.

[27] Smith, R. *Inhibition: History and Meaning in the Sciences of Mind and Brain* (University of California Press, 1992).

[28] Potvin P, Cyr G (2017). Toward a durable prevalence of scientific conceptions: tracking the effects of two interfering misconceptions about buoyancy from preschoolers to science teachers. J. Res. Sci. Teach..

[29] Goldberg RF, Thompson-Schill SL (2009). Developmental “roots” in mature biological knowledge. Psychol. Sci..

[30] Lewis EL, Linn MC (1994). Heat energy and temperature concepts of adolescents, adults, and experts: Implications for curricular improvements. J. Res. Sci. Teach..

[31] Kozhevnikov M, Hegarty M (2001). Impetus beliefs as default heuristics: dissociation between explicit and implicit knowledge about motion. Psychon. Bull. Rev..

[32] Fugelsang JA, Dunbar KN (2005). Brain-based mechanisms underlying complex causal thinking. Neuropsychologia.

[33] Mareschal D (2016). The neuroscience of conceptual learning in science and mathematics. Curr. Opin. Behav. Sci..

[34] Brault Foisy L-M, Potvin P, Riopel M, Masson S (2015). Is inhibition involved in overcoming a common physics misconception in mechanics?. Trends Neurosci. Educ..

[35] Dunbar, K. N. & Stein, C. In *Thinking with Data*, (eds. Lovett, M. C. & Shah, P.) 193–206 (Lawrence ErlbaumAssociates, 2007).

[36] Masson S, Potvin P, Riopel M, Brault Foisy L-M (2014). Differences in brain activation between novices and experts in science during a task involving a common misconception in electricity. Mind, Brain, Educ..

[37] Potvin P, Malenfant-Robichaud G, Cormier C, Masson S (2020). Coexistence of misconceptions and scientific conceptions in chemistry professors: a mental chronometry and fMRI study. Front. Educ.

[38] Aron AR (2007). The neural basis of inhibition in cognitive control. Neuroscientist.

[39] Aron AR, Robbins TW, Poldrack RA (2014). Inhibition and the right inferior frontal cortex: one decade on. Trends Cogn. Sci..

[40] Badre D, Wagner AD (2007). Left ventrolateral prefrontal cortex and the cognitive control of memory. Neuropsychologia.

[41] Danker JF, Gunn P, Anderson JR (2008). A rational account of memory predicts left prefrontal activation during controlled retrieval. Cereb. Cortex.

[42] Brust JCM (2007). The human frontal lobes: functions and disorders. Neurologist.

[43] MacDonald M, Cohen JD, Stenger V, Carter CS (2000). Dissociating the role of the dorsolateral prefrontal cortex and anterior cingulate cortex in cognitive control. Science.

[44] Mylius V (2013). Definition of DLPFC and M1 according to anatomical landmarks for navigated brain stimulation: Inter-rater reliability, accuracy, and influence of gender and age. NeuroImage.

[45] Vassena E, Holroyd CB, Alexander WH (2017). Computational models of anterior cingulate cortex: at the crossroads between prediction and effort. Front. Neurosci.

[46] Allaire-Duquette G, Bélanger M, Grabner RH, Koschutnig K, Masson S (2019). Individual differences in science competence among students are associated with ventrolateral prefrontal cortex activation. J. Neurosci. Res..

[47] Diamond A (2013). Executive functions. Annu. Rev. Psychol..

[48] Borst G, Poirel N, Pineau A, Cassotti M, Houdé O (2013). Inhibitory control efficiency in a Piaget-like class-inclusion task in school-age children. Dev. Psychol..

[49] De Neys, W. On dual and single process models of thinking. *Perspect. Psychol. Sci*. https://hal.archives-ouvertes.fr/hal-03025509 (in press). 10.1177/1745691620964172 33621468

[50] Bago B, De Neys W (2020). Advancing the specification of dual process models of higher cognition: a critical test of the hybrid model view. Think. Reasoning.

[51] Potvin P, Turmel E, Masson S (2014). Linking neuroscientific research on decision-making to the educational context of novice students assigned to a multiple-choice scientific task involving common misconceptions about electrical circuits. Front. Hum. Neurosci.

[52] Baddeley AD, Hitch G (1993). The recency effect: Implicit learning with explicit retrieval?. Mem. Cognition.

[53] Lanoë C, Vidal J, Lubin A, Houdé O, Borst G (2016). Inhibitory control is needed to overcome written verb inflection errors: Evidence from a developmental negative priming study. Cogn. Dev..

[54] Dempster F, Corkill A (1999). Interference and inhibition in cognition and behavior: comments on the commentaries. Educ. Psychol. Rev..

[55] Houdé O, Borst G (2014). Measuring inhibitory control in children and adults: Brain imaging and mental chronometry. Front. Psychol.

[56] Shtulman A, Legare CH (2020). Competing explanations of competing explanations: accounting for conflict between scientific and folk explanations. Top. Cogn. Sci..

[57] Bush G, Whalen PJ, Shin LM, Rauch SL (2006). The counting Stroop: a cognitive interference task. Nat. Protoc..

[58] Lemire-Rodger, S. et al. Inhibit, switch, and update: a within-subject fMRI investigation of executive control. *Neuropsychologi.* **132**, 107134, 10.1016/j.neuropsychologia.2019.107134 (2019). 10.1016/j.neuropsychologia.2019.107134 31299188

[59] Shulman GL (2009). Interaction of stimulus-driven reorienting and expectation in ventral and dorsal frontoparietal and basal ganglia-cortical networks. J. Neurosci..

[60] Doricchi F, MacCi E, Silvetti M, MacAluso E (2010). Neural correlates of the spatial and expectancy components of endogenous and stimulus-driven orienting of attention in the posner task. Cereb. Cortex..

[61] Swick D, Ashley V, Turken AU (2008). Left inferior frontal gyrus is critical for response inhibition. BMC Neurosci.

[62] Cai W, Ryali S, Chen T, Li C-SR, Menon V (2014). Dissociable roles of right inferior frontal cortex and anterior insula in inhibitory control: evidence from intrinsic and task-related functional parcellation, connectivity, and response profile analyses across multiple datasets. J. Neurosci..

[63] Levy BJ, Wagner AD (2012). Cognitive control and right ventrolateral prefrontal cortex: reflexive reorientation, motor inhibition, and action updating. Ann. N. Y. Acad. Sci..

[64] Houdé O, Borst G (2015). Evidence for an inhibitory-control theory of the reasoning brain. Front. Hum. Neurosci..

[65] Collette F (2001). The functional anatomy of inhibition processes investigated with the Hayling task. Neuroimage.

[66] Grosselin A (2010). Inhibition des réponses automatiques au test du Hayling dans la schizophrénie. Encephale.

[67] Nathaniel-James DA, Fletcher P, Frith CD (1997). The functional anatomy of verbal initiation and suppression using the Hayling Test. Neuropsychologia.

[68] Hebb, D. O. *The Organization of Behavior: A Neuropsychological Theory* (Wiley, 1949).

[69] Vaughn AR, Brown RD, Johnson ML (2020). Understanding conceptual change and science learning through educational neuroscience. Mind Brain Educ..

[70] Cunnington R, Windischberger C, Moser E (2005). Premovement activity of the pre-supplementary motor area and the readiness for action: Studies of time-resolved event-related functional MRI. Hum. Mov. Sci..

[71] Rogers BP, Carew JD, Meyerand ME (2004). Hemispheric asymmetry in supplementary motor area connectivity during unilateral finger movements. NeuroImage.

[72] Simmonds D, Pekar JJ, Mostofsky SH (2008). Meta-analysis of Go/No-go tasks demonstrating that fMRI activation associated with response inhibition is task-dependent. Neuropsychologia.

[73] Aron AR, Behrens TE, Smith S, Frank MJ, Poldrack RA (2007). Triangulating a cognitive control network using diffusion-weighted magnetic resonance imaging (MRI) and functional MRI. J. Neurosci..

[74] Rottschy C (2012). Modelling neural correlates of working memory: a coordinate-based meta-analysis. NeuroImage.

[75] Vorobyev VA (2004). Linguistic processing in visual and modality-nonspecific brain areas: PET recordings during selective attention. Cogn. Brain Res..

[76] Dehaene S, Cohen L (2011). The unique role of the visual word form area in reading. Trends Cogn. Sci..

[77] Ghosh S, Basu A, Kumaran SS, Khushu S (2010). Functional mapping of language networks in the normal brain using a word-association task. Indian J. Radiol. Imaging.

[78] Renier LA (2010). Preserved functional specialization for spatial processing in the middle occipital gyrus of the early blind. Neuron.

[79] Neubauer AC, Fink A (2009). Intelligence and neural efficiency. Neurosci. Biobehav. Rev..

[80] Vossel S, Geng JJ, Fink GR (2014). Dorsal and ventral attention systems: distinct neural circuits but collaborative roles. Neuroscientist.

[81] Indovina I, MacAluso E (2007). Dissociation of stimulus relevance and saliency factors during shifts of visuospatial attention. Cereb. Cortex.

[82] Botvinick MM (2007). Conflict monitoring and decision-making: reconciling two perspectives on anterior cingulate function. Cogn., Affect. Behav. Neurosci..

[83] Eijsker N, Schröder A, Smit DJA, van Wingen G, Denys D (2019). Neural basis of response bias on the stop signal task in misophonia. Front. Psychiatry.

[84] Hu S, Ide JS, Zhang S, Li CSR (2016). The right superior frontal gyrus and individual variation in proactive control of impulsive response. J. Neurosci..

[85] Bhattacharjee S (2020). The role of primary motor cortex: more than movement execution. J. Mot. Behav..

[86] Poldrack RA (2006). Can cognitive processes be inferred from neuroimaging data?. Trends Cogn. Sci..

[87] Yarkoni T, Poldrack RA, Nichols TE, Van Essen DC, Wager TD (2011). Large-scale automated synthesis of human functional neuroimaging data. Nat. methods.

[88] Kahneman, D. In *Neuroeconomics: Decision Making and the Brain*, (eds. Glimcher, P. W., Camerer, C. F., Fehr, E. & Poldrack, R. A.) (Elsevier, 2009).

[89] Hutzler F (2014). Reverse inference is not a fallacy per se: cognitive processes can be inferred from functional imaging data. Neuroimage.

[90] Shiffrin RM, Schneider W (1977). Controlled and automatic human information processing: II. Perceptual learning, automatic attending and a general theory. Psychol. Rev..

[91] Shiffrin, R. M. & Dumais, S. T. In *The Development of Automatism*, (ed. Anderson, J. R.) (Psychology Press, 1981).

[92] Elio R (1986). Representation of similar well‐learned cognitive procedures. Cogn. Sci..

[93] Potvin P, Masson S, Lafortune S, Cyr G (2014). Persistence of the intuitive conception that heavier objects sink more: a reaction time study with different levels of interference. Int. J. Sci. Math. Educ..

[94] Johnson CN, Harris PL (1994). Magic: special but not excluded. Br. J. Dev. Psychol..

[95] National Institute of Mental Health. Inclusion of women and minorities as participants in research involving human subjects. https://grants.nih.gov/grants/funding/women_min/women_min.htm (2019).

[96] Canadian Association of University Teachers. 2013/2014 CAUT Almanac of Post-Secondary Education in Canada. 80. http://www.caut.ca/docs/default-source/almanac/almanac_2013-2014_print_finalE20A5E5CA0EA6529968D1CAF.pdf?sfvrsn=2 (2014).

[97] Flesch R (1948). “A new readability yardstick”. J. Appl. Psychol..

[98] Kandel L, Moles A (1958). Application de l’indice de Flesch à la langue française. Cah. Études de. Radio-TéléVis..

[99] AAAS [American Association for the Advancement of Science]. Misconception EGM021: cold/frozen objects do not have any thermal energy. AAAS Project 2061. http://assessment.aaas.org/misconceptions/0/EGM021/216 (n.d.).

[100] Wellcome Trust Center for Neuroimaging. Statistical parametric mapping. http://www.fil.ion.ucl.ac.uk/spm/software/ (2009).

[101] Penny, W. & Henson, R. N. In *Statistical Parametric Mapping* (eds. Friston, K., Ashburner, J., Kiebel, S., Nichols, T. & Penny, W.) (Elsevier, 2006).

[102] Woo CW, Krishnan A, Wager TD (2014). Cluster-extent based thresholding in fMRI analyses: pitfalls and recommendations. NeuroImage.

[103] Friston KJ, Worsley KJ, Frackowiak RSJ, Mazziotta JC, Evans AC (1994). Assessing the significance of focal activations using their spatial extent. Hum. Brain Mapp..

[104] Smith SM, Nichols TE (2009). Threshold-free cluster enhancement: addressing problems of smoothing, threshold dependence and localisation in cluster inference. NeuroImage.

[105] Friston, K. in *Brain Mapping: The Disorders* (eds. Mazziotta, J. C., Toga, A. W. & Frackowiak, R. S. J.) (Academic, 2000).

[106] Wager TD, Lindquist M, Kaplan L (2007). Meta-analysis of functional neuroimaging data: current and future directions. Soc. Cogn. Affect. Neurosci..

[107] Rorden C, Karnath HO, Bonilha L (2007). Improving lesion-symptom mapping. J. Cogn. Neurosci..

