# Supplementary material for: An fMRI study of scientists with a Ph.D. in physics confronted with naive ideas in science
Source: NPJ Sci Learn. 2021 May 11;6:11. doi: 10.1038/s41539-021-00091-x (PMC8113248; doi:10.1038/s41539-021-00091-x)
Supplement: Supplementary file 1 — Reporting Summary [file 41539_2021_91_MOESM1_ESM.pdf]

nature research

Corresponding author(s): Genevieve Allaire-Duquette

Last updated by author(s): Mar 31, 2021

Reporting Summary

Nature Research wishes to improve the reproducibility of the work that we publish. This form provides structure for consistency and transparency in reporting. For further information on Nature Research policies, see our Editorial Policies and the Editorial Policy Checklist.

Statistics

For all statistical analyses, confirm that the following items are present in the figure legend, table legend, main text, or Methods section.

n/a	Confirmed	
☐	☒	The exact sample size (n) for each experimental group/condition, given as a discrete number and unit of measurement
☒	☐	A statement on whether measurements were taken from distinct samples or whether the same sample was measured repeatedly
☐	☒	The statistical test(s) used AND whether they are one- or two-sided<br>*Only common tests should be described solely by name; describe more complex techniques in the Methods section.*
☒	☐	A description of all covariates tested
☐	☒	A description of any assumptions or corrections, such as tests of normality and adjustment for multiple comparisons
☐	☒	A full description of the statistical parameters including central tendency (e.g. means) or other basic estimates (e.g. regression coefficient) AND variation (e.g. standard deviation) or associated estimates of uncertainty (e.g. confidence intervals)
☐	☒	For null hypothesis testing, the test statistic (e.g. F, t, r) with confidence intervals, effect sizes, degrees of freedom and P value noted<br>*Give P values as exact values whenever suitable.*
☒	☐	For Bayesian analysis, information on the choice of priors and Markov chain Monte Carlo settings
☒	☐	For hierarchical and complex designs, identification of the appropriate level for tests and full reporting of outcomes
☐	☒	Estimates of effect sizes (e.g. Cohen's d, Pearson's r), indicating how they were calculated

Our web collection on statistics for biologists contains articles on many of the points above.

Software and code

Policy information about availability of computer code

Data collection	Stimuli presentation was accomplished with E-Prime 2.0 software (Psychology Software Tools, inc.). Participants' responses were collected with a Fiber Optic Button Response System (Psychological Software Tools, inc., Sharpsburg, Pennsylvania, USA).
Data analysis	Analysis of structural and functional imaging of the functional magnetic resonance image (fMRI) data was performed using open source SPM8 software v6313 (Wellcome Trust Center for Neuroimaging, 2009). Behavioral data analysis was performed using SPSS version 27.0 (SPSS Inc., Chicago, IL, USA).

For manuscripts utilizing custom algorithms or software that are central to the research but not yet described in published literature, software must be made available to editors and reviewers. We strongly encourage code deposition in a community repository (e.g. GitHub). See the Nature Research guidelines for submitting code & software for further information.

Data

Policy information about availability of data

All manuscripts must include a data availability statement. This statement should provide the following information, where applicable:

- Accession codes, unique identifiers, or web links for publicly available datasets
- A list of figures that have associated raw data
- A description of any restrictions on data availability

The data used in the analysis can't be made available to researchers in an openly available repository according to the current legal provisions of Quebec (Canada) and in particular the Act respecting the protection of personal information in the private sector (L.R.C. (1985), ch. P-21, http://www.legisquebec.gouv.qc.ca/en/showdoc/cs/P-39.1). The MATLAB script used to automate fMRI data preprocessing and statistical analysis is available from the corresponding author, upon reasonable request for purposes of reproducing or extending the analysis.

Field-specific reporting

Please select the one below that is the best fit for your research. If you are not sure, read the appropriate sections before making your selection.

☐ Life sciences ☒ Behavioural & social sciences ☐ Ecological, evolutionary & environmental sciences

For a reference copy of the document with all sections, see nature.com/documents/nr-reporting-summary-flat.pdf

Behavioural & social sciences study design

All studies must disclose on these points even when the disclosure is negative.

Study description	Neuroimaging study of scientists with a PhD in physics assessing the scientific value of naïve ideas related to their domain of expertise (physics) and to a domain in which they have a more basic level of expertise (biology).
Research sample	25 scientists with a PhD in physics from urban area in Canada
Sampling strategy	Invitations were sent to all physics and engineering departments of the greater Montreal area in Canada. Desmond & Glover (2002) suggest that about 24 subjects are required to achieve 80% power after correcting for multiple comparisons.
Data collection	Stimuli presentation was accomplished with E-Prime 2.0 software (Psychology Software Tools, inc.). Participants' responses were collected with a Fiber Optic Button Response System (Psychological Software Tools, inc., Sharpsburg, Pennsylvania, USA). Participant was alone in the scanner room during data collection.
Timing	The total experiment time was about 20 min. Before the MRI session, a practice task comprising different statements was performed on a desktop computer and in a mock scanner to allow habituation in an environment less daunting than a real scanner.
Data exclusions	Only right-handed participants with MRI compatible metallic implants located lower than the neck were considered.
Non-participation	A total of 518 invitations were sent by email. 356 of them never responded. 112 potential participants declined for various reasons (too far to drive to the scanner, no time, doens't speak French well enough or doesn't have a Ph.D., were on a sabbatical leave). 38 agreed to participate, but 13 dropped during the process mostly because of a lack of availability when came the time to schedule the fMRI session.
Randomization	Participants were not allocated to experimental groups.

Reporting for specific materials, systems and methods

We require information from authors about some types of materials, experimental systems and methods used in many studies. Here, indicate whether each material, system or method listed is relevant to your study. If you are not sure if a list item applies to your research, read the appropriate section before selecting a response.

Materials & experimental systems

n/a	Involved in the study	
☒	☐	Antibodies
☒	☐	Eukaryotic cell lines
☒	☐	Palaeontology and archaeology
☒	☐	Animals and other organisms
☐	☒	Human research participants
☒	☐	Clinical data
☒	☐	Dual use research of concern

Methods

n/a	Involved in the study	
☒	☐	ChIP-seq
☒	☐	Flow cytometry
☐	☒	MRI-based neuroimaging

Human research participants

Policy information about studies involving human research participants

Population characteristics	25 right-handed adults who complete a PhD in physics, including 23 male and 2 female participants (age range 28-60 y, $\bar{x}$ = 45 y). Participants reported no abnormal neurological history.
Recruitment	Invitations were sent by email to all physics and engineering departments of the greater Montreal area in Canada.
Ethics oversight	Research Ethics Board of Quebec's Neuroimaging Network (CMER RNQ 13-14-023) endorsed by the Ministry of Health and Social Services of Quebec (Canada).

Note that full information on the approval of the study protocol must also be provided in the manuscript.

Magnetic resonance imaging

Experimental design

Design type	block design
Design specifications	The experiment was presented in a block design divided into 4 runs of 10 blocks comprising 4 statements each and presented in a random sequence. The trials were presented until the participant responded, or for a maximum duration of 10 s. A fixation cross was displayed for 15 s between each block.
Behavioral performance measures	Correct buttom press and response time were recorded. All participants performed above chance level (50%) (min = 73%).

Acquisition

Imaging type(s)	functional and structural
Field strength	3-Tesla (Siemens Prisma)
Sequence & imaging parameters	Functional images were obtained with a single-shot gradient echo EPI sequence sensitive to blood oxygen level-dependent (BOLD) contrast (TR = 2000 ms, TE = 30 ms, FA = 90°, FOV = 192 mm, matrix size = 64 × 64, pixel size = 3 × 3 mm, interleaved). Thirty-three 3-mm-thick transverse slices with a distance factor of 25 % were acquired parallel to the AC–PC line. For each run, between 120 and 200 functional volumes were obtained. The first two volumes were discarded to account for T1 saturation effects. Structural images were obtained using a T1-weighted 3D MPRAGE sequence (TR = 2,300 ms, TI = 900 ms, TE = 2.26 ms, FA = 9°, FOV = 256 mm, matrix size = 256 × 256, 1 slab, 176 images per slab, pixel spacing = 1 × 1 mm). The acquisition of structural images lasted about 10 min
Area of acquisition	whole brain, FOV = 192 mm

Diffusion MRI ☐ Used ☒ Not used

Preprocessing

Preprocessing software	The functional data of each participant were motion-corrected, co-registered with structural data, and then spatially normalized into the standard MNI space (Montreal Neurological Institute). Head motion was corrected with SPM8 across and within sessions of every individual subject. The fMRI series were corrected for motion using the standard SPM "spm_realign" procedure and mean images were calculated. Images are realigned to the mean image.
Normalization	Normalization was performed using the standard SPM "spm_coreg" procedure that co-registers the individuals T1 to their EPI and allows for a better normalization to the MNI template. The "single generative model" segmentation approach was employed to determine normalization parameters and used to normalize spatially the realigned images to the MNI reference space. This model involves alternates among classification, bias correction, and registration steps. The normalized EPI images were smoothed with a Gaussian kernel of 8 mm FWHM.
Normalization template	standard MNI space (Montreal Neurological Institute)
Noise and artifact removal	For each participant, fMRI images were high-pass filtered at 128 s to remove low-frequency drifts.
Volume censoring	No motion correction was performed. Data was manually inspected and no motion "spikes" were identified.

Statistical modeling & inference

Model type and settings	univariate. the statistical analysis was conducted using the general linear model. Model time courses for each experimental condition block were generated on the basis of the hemodynamic response function implemented in SPM8. The analysis for the entire group was performed by computing linear t-contrasts (experimental conditions vs. fixation period) for each subject individually, which were then entered into random effects (second-level) whole-brain ANOVA using full factorial design analysis in SPM8.
Effect(s) tested	A within-within-subject analysis of variance (ANOVA) with two within subject factors of two levels each: EXPERTISE (basic vs. advanced) x CONGRUENCY (congruent vs. incongruent) and subsequent t-tests to determine the direction of the effects.

Specify type of analysis: ☐ Whole brain ☐ ROI-based ☒ Both

Anatomical location(s)	Frontal lobe and bilateral anterior cingulate cortex using AAL
Statistic type for inference (See Eklund et al. 2016)	The ANOVA results are reported at a cluster-wise familywise error (FWE) corrected threshold pFWE-CORRECTED < .05 across the whole brain, using a primary voxelwise threshold of pUNCORRECTED < .005. Post-hoc t-contrasts revealing the direction of the main effect and interaction (ANOVA) are reported using voxelwise threshold pUNCORRECTED < 0.005 to account for their lower statistical power compared to ANOVAs.
Correction	FWE for ANOVA results, and reduced threshold of pUNCORRECTED < 0.005 for t-tests

Models & analysis

n/a	Involved in the study
☒	☐ Functional and/or effective connectivity
☒	☐ Graph analysis
☒	☐ Multivariate modeling or predictive analysis
