# Supplementary material for: An fMRI study of scientists with a Ph.D. in physics confronted with naive ideas in science
Source: NPJ Sci Learn. 2021 May 11;6:11. doi: 10.1038/s41539-021-00091-x (PMC8113248; doi:10.1038/s41539-021-00091-x)
Supplement: Supplementary file 2 — Supplementary Information [file 41539_2021_91_MOESM2_ESM.pdf]

Supplementary Methods

Scientific Statements Task
Items translated freely from French into English (original items between brackets).

Physics

Pair #	Scientific Value	Congruency	Statement	Naïve Ideas
1	T	Congruent	Magnets exert a pull on an iron rod. (Les aimants attirent une tige de fer.)	Magnets exert a pull on all metals. [1]
1’	F	Incongruent	Magnets exert a pull on an aluminum rod. (Les aimants attirent une tige d’aluminium.)	
2	T	Congruent	A flow of electrons produces an electric current. (Un déplacement d'électrons produit un courant électrique.)	Electricity is only produces by a flow of electrons. [2]
2’	T	Incongruent	A flow of protons produces an electric current. (Un déplacement de protons produit un courant électrique.)	
3	T	Congruent	A campfire contains thermal energy. (Un feu de camp contient de l'énergie thermique.)	Only hot objects contain thermal energy. [3]
3’	T	Incongruent	An ice cube contains thermal energy. (Un cube de glace contient de l'énergie thermique.)	
4	T	Congruent	The Earth exerts a gravitational pull on pebbles. (La Terre attire les cailloux.)	The more massive bodies exert gravity. [4]
4’	T	Incongruent	Pebbles exert a gravitational pull on the Earth. (Les cailloux attirent la Terre.)	
5	F	Congruent	A light-year is a measure of mass. (Une année-lumière est une unité de mesure de la masse.)	A light-year is a measure of time. [5]
5’	T	Incongruent	A light-year is a measure of distance. (Une année-lumière est une unité de mesure de la distance.)	
6	F	Congruent	Gravitational attraction is the same on the Moon as on the Earth. (La force gravitationnelle est la même sur la Lune que sur la Terre.)	Gravity is weaker underwater. [6,7]
6’	T	Incongruent	Gravitational attraction is the same underwater as on mainland. (La force gravitationnelle est la même sous l'eau que sur la terre ferme.)	
7	F	Congruent	The Sun revolves around the Moon. (Le Soleil tourne autour de la Lune.)	The Moon only revolves around Earth. [8]
7’	T	Incongruent	The Moon revolves around the Sun. (La Lune tourne autour du Soleil.)	
8	T	Congruent	The Solar system comprises many planets. (Le système solaire contient plusieurs planètes.)	The Solar system comprises many stars scattered in space. [9]
8’	F	Incongruent	The Solar system comprises many stars. (Le système solaire contient plusieurs étoiles.)	
9	T	Congruent	The Moon revolves around the Earth. (La Lune tourne autour de la Terre.)	Night-day cycle is caused by the Sun revolving around the Earth. [10]
9	F	Incongruent	The Sun revolves around the Earth. (Le Soleil tourne autour de la Terre.)	
10	F	Congruent	The Moon is a star. (La Lune est une étoile.)	The Sun is not a star. [11]
10’	T	Incongruent	The Sun is a star. (Le Soleil est une étoile.)	

Physics

Pair #	Scientific Value	Congruency	Statement	Naïve Ideas
11	F	Congruent	Boiling water contains helium bubbles.<br>(Les bulles de gaz qui se forment dans l'eau en ébullition sont faites d'hélium.)	Bubbles in boiling liquids are made of air. [12]
11’	T	Incongruent	Boiling water contains water bubbles.<br>(Les bulles de gaz qui se forment dans l'eau en ébullition sont faites d'eau.)	
12	F	Congruent	When an ice cube melts, the water decomposes.<br>(Lorsqu’un cube de glace fond, l’eau se décompose.)	When temperature increases, volume increases. [13]
12’	T	Incongruent	When a cube of ice melts, the water contracts.<br>(Lorsqu’un cube de glace fond, l’eau se contracte.)	
13	F	Congruent	Boiling water contains nitrogen and helium bubbles.<br>(L'eau qui bout contient des bulles d'azote et d'hélium.)	Bubbles in boiling liquids are made of O_2 and N_2. [14]
13’	F	Incongruent	Boiling water contains oxygen and hydrogen bubbles.<br>(L'eau qui bout contient des bulles d'oxygène et d'hydrogène.)	
14	T	Congruent	Liquid water is denser than steam.<br>(L'eau liquide est plus dense que la vapeur d'eau.)	Solid substances are denser than liquid substances. [15]
14’	F	Incongruent	Frozen water is denser than liquid water.<br>(L'eau gelée est plus dense que l'eau liquide.)	
15	T	Congruent	Heating warm water increases its temperature.<br>(Chauffer une casserole d'eau tiède augmente sa température.)	The temperature varies during a phase change. [16]
15’	F	Incongruent	Heating boiling water increases its temperature.<br>(Chauffer une casserole d'eau bouillante augmente sa température.)	
16	T	Congruent	Light waves travel in vacuum.<br>(Les ondes lumineuses voyagent dans le vide.)	Sound can travel in vacuum. [17]
16’	F	Incongruent	Sound waves travel in vacuum.<br>(Les ondes sonores voyagent dans le vide.)	
17	T	Congruent	Light waves travel faster than sound waves.<br>(Les ondes lumineuses voyagent plus vite que les ondes sonores.)	Radio waves and X-Rays vary according to speed. [9]
17’	F	Incongruent	Radio waves and X-Rays travel at different speeds.<br>(Les ondes radio et les rayons-X voyagent à différentes vitesses.)	
18	F	Congruent	One gram of Styrofoam is heavier than one gram of iron.<br>(Un gramme de styromousse est plus massif qu'un gramme de fer.)	Dense substances are more massive. [6,18]
18’	F	Incongruent	One gram of iron is heavier than one gram of Styrofoam.<br>(Un gramme de fer est plus massif qu'un gramme de styromousse.)	
19	T	Congruent	An astronaut has the same volume on Earth as on the Moon.<br>(Un astronaute possède le même volume sur la Terre que sur la Lune.)	Mass and weight are identical. [6,19]
19’	T	Incongruent	An astronaut has the same mass on Earth as on the Moon.<br>(Un astronaute possède la même masse sur la Terre que sur la Lune.)	
20	T	Congruent	Gases expand when they get warmer.<br>(Les gaz se dilatent lorsqu’il fait plus chaud.)	Solids do not expand. [20,21]
20’	T	Incongruent	Concrete expands when it gets warmer.<br>(Le béton se dilate lorsqu'il fait plus chaud.)	

Physics

Pair #	Scientific Value	Congruency	Statement	Naïve Ideas
21	F	Congruent	Without the moon, there would be no seasons on Earth.<br>(Sans la Lune, il n'y aurait pas de saisons sur la Terre.)	The tides on Earth are solely caused by the action of the Moon. [9]
21'	F	Incongruent	Without the moon, there would be no tides on Earth.<br>(Sans la Lune, il n'y aurait pas de marées sur la Terre.)	
22	F	Congruent	Seasons result from the variation of the distance between the Earth and Mars.<br>(La variation de la distance entre la Terre et Mars cause les saisons.)	The variation of the distance between the Earth and the Sun causes the seasons. [22]
22'	F	Incongruent	Seasons result from the variation of the distance between the Earth and the Sun.<br>(La variation de la distance entre la Terre et le Soleil cause les saisons.)	
23	F	Congruent	The Earth rotates around its axis in one season.<br>(La Terre tourne autour de son axe en une saison.)	The Earth revolves around its axis in one year. [9]
23'	F	Incongruent	The Earth rotates around its axis in one year.<br>(La Terre tourne autour de son axe en une année.)	
24	F	Congruent	A car traveling at 50 km / h has the same kinetic energy as a bike traveling at 10 km / h.<br>(Une voiture roulant à 50 km/h a la même énergie cinétique qu'un vélo roulant à 10 km/h.)	The amount of kinetic energy depends only on speed. [23]
24'	F	Incongruent	A car traveling at 10 km / h has the same kinetic energy as a bike traveling at 10 km / h.<br>(Une voiture roulant à 10 km/h a la même énergie cinétique qu'un vélo roulant à 10 km/h.)	
25	F	Congruent	A glass of water at 50 ° C contains as much heat energy as a drop of water at 25 ° C.<br>(Un verre d'eau à 50°C contient autant d'énergie thermique qu'une goutte d'eau à 25°C.)	Two bodies at the same temperature have the same heat. [24]
25'	F	Incongruent	A glass of water at 25 ° C contains as much heat energy as a drop of water at 25 ° C.<br>(Un verre d'eau à 25°C contient autant d'énergie thermique qu'une goutte d'eau à 25°C.)	
26	T	Congruent	A lit candle emits infrared radiation.<br>(Une chandelle allumée émet un rayonnement infrarouge.)	Only hot objects emit infrared radiation. [25]
26'	T	Incongruent	An extinct candle emits infrared radiation.<br>(Une chandelle éteinte émet un rayonnement infrarouge.)	
27	F	Congruent	Light waves and radio waves are gases.<br>(La lumière et les ondes radio sont gazeuses.)	Radio waves and light waves are of different nature. [9,26]
27'	T	Incongruent	Light waves and radio waves are of the same nature.<br>(La lumière et les ondes radio sont de même nature.)	
28	F	Congruent	Radio waves are visible.<br>(Les ondes radio sont des ondes visibles.)	Radio waves are sound waves. [27]
28'	F	Incongruent	Radio waves are audible.<br>(Les ondes radio sont des ondes audibles.)	
29	T	Congruent	The Earth exerts a gravitational pull on the nacelle of a balloon.<br>(La Terre attire la nacelle d'une montgolfière.)	The gases are not subject to gravity. [28]
29'	T	Incongruent	The Earth exerts a gravitational pull on the hot air of a balloon.<br>(La Terre attire l'air chaud d'une montgolfière.)	
30	T	Congruent	The Earth exerts a gravitational pull on a book falling off a shelf.<br>(Un livre qui tombe d'une étagère est attiré par la Terre.)	Stationary objects are not subject to gravity. [29]
30'	T	Incongruent	The Earth exerts a gravitational pull on a book placed on a shelf.<br>(Un livre posé sur une étagère est attiré par la Terre.)	

Physics

Pair #	Scientific Value	Congruency	Statement	Naïve Ideas
31	F	Congruent	1 mL of glass contains the same amount of substance than 2 mL of glass.<br>(1 mL de verre comprend la même quantité de matière que 2 mL de verre.)	Both mass and volume measures reflect the amount of matter. [30]
31’	F	Incongruent	1 mL of glass contains the same amount of substance as 1 mL of iron.<br>(1 mL de verre comprend la même quantité de matière que 1 mL de fer.)	
32	T	Congruent	An iron cube contains atoms.<br>(Un cube de fer contient des atomes.)	There is no space between particles. [31]
32’	T	Incongruent	An iron cube contains a vacuum.<br>(Un cube de fer contient du vide.)	

Summary (physics statements)

Condition	Congruent		Incongruent	
Scientific response	True	False	True	False
	16	16	16	16

Biology

Pair #	Scientific Value	Congruency	Statement	Naïve Ideas
1	F	Congruent	Cacti are animals.<br>(Les cactus sont des animaux.)	Arachnids and insects are not animals. [32]
1’	T	Incongruent	Spiders are animals.<br>(Les araignées sont des animaux.)	
2	F	Congruent	Whales are reptiles.<br>(Les baleines sont des reptiles.)	Whales are big fish. [33]
2’	F	Incongruent	Whales are fish.<br>(Les baleines sont des poissons.)	
3	F	Congruent	Whales are genetically closer to plants than to fish.<br>(Les baleines sont génétiquement plus proches des plantes que des poissons.)	Whales are big fish. [33]
3’	F	Incongruent	Whales are genetically closer to fish than humans.<br>(Les baleines sont génétiquement plus proches des poissons que des humains.)	
4	T	Congruent	Gorillas are animals.<br>(Les gorilles sont des animaux.)	Humans are not animals. [34]
4’	T	Incongruent	Human beings are animals.<br>(Les humains sont des animaux.)	
5	F	Congruent	Gorillas are invertebrates.<br>(Les gorilles sont des invertébrés.)	Animals that have joints are vertebrates. [35]
5’	T	Incongruent	Lobsters are invertebrates.<br>(Les homards sont des invertébrés.)	
6	F	Congruent	Horses are invertebrates.<br>(Les chevaux sont des invertébrés.)	Animals that have joints are vertebrates. [35]
6’	F	Incongruent	Snakes are invertebrates.<br>(Les serpents sont des invertébrés.)	
7	F	Congruent	Stones are biological tissues.<br>(Les pierres sont des tissus biologiques.)	Only blood and muscles are biological tissues. [36]
7’	T	Incongruent	Bones are biological tissues.<br>(Les os sont des tissus biologiques.)	
8	F	Congruent	Air is comprised of cells.<br>(L’air est formé de cellules)	Air is comprised of cells. [36]
8’	F	Incongruent	Water is comprised of cells.<br>(L'eau est formée de cellules.)	
9	T	Congruent	Cells are bigger than chromosomes.<br>(Les cellules sont plus grosses que les chromosomes.)	Proteins are molecules that are bigger than cells. [37]
9	F	Incongruent	The proteins are bigger than cells.<br>(Les protéines sont plus grosses que les cellules.)	
10	T	Congruent	Egg cells contain chromosomes.<br>(Les ovules contiennent des chromosomes.)	Chromosomes are only found in gametes. [38]
10’	T	Incongruent	Neurons contain chromosomes.<br>(Les neurones contiennent des chromosomes.)	

Biology

Pair #	Scientific Value	Congruency	Statement	Naïve Ideas
11	F	Congruent	Chlorophyll can be a source of energy for animal cells.<br>(La chlorophylle peut être une source d'énergie pour les cellules animales.)	Proteins are only used to build or repair biological tissue. [39]
11'	T	Incongruent	Proteins can be a source of energy for animal cells.<br>(Les protéines peuvent être une source d'énergie pour les cellules animales.)	
12	T	Congruent	The respiration of plants takes place at night.<br>(La respiration des plantes a lieu la nuit.)	The respiration of plants takes place only at night. [40]
12'	T	Incongruent	The respiration of plants takes place during the day.<br>(La respiration des plantes a lieu le jour.)	
13	T	Congruent	Plants emit oxygen gas.<br>(Les plantes émettent de l'oxygène gazeux.)	Plants only do photosynthesis. [41]
13'	T	Incongruent	Plants emit carbon dioxide gas.<br>(Les plantes émettent du gaz carbonique.)	
14	T	Congruent	Green-leaved plants do photosynthesis.<br>(Les plantes aux feuilles vertes font de la photosynthèse.)	Chlorophyll is the only photosynthetic pigment. [42]
14'	T	Incongruent	Red-leaved plants do photosynthesis.<br>(Les plantes aux feuilles rouges font de la photosynthèse.)	
15	T	Congruent	Cellular respiration transforms oxygen.<br>(La respiration cellulaire implique de l'oxygène.)	Respiration does not involve energy conversion (only substance conversion). [43]
15'	T	Incongruent	Cellular respiration transforms energy.<br>(La respiration cellulaire transforme de l'énergie.)	
16	T	Congruent	Insects are alive.<br>(Les insectes sont vivants.)	What is moving is alive. [44]
16'	F	Incongruent	Campfires are alive.<br>(Un feu de camp est vivant)	
17	T	Congruent	Cells are alive.<br>(Les cellules sont vivantes.)	Nuts and seeds are only alive once sown. [44]
17'	T	Incongruent	Nuts are alive.<br>(Les noix sont vivantes.)	
18	F	Congruent	Stones are part of the food chain.<br>(Les pierres font partie de la chaine alimentaire.)	Only animals are part of the food chain. [45]
18'	T	Incongruent	Plants are part of the food chain.<br>(Les plantes font partie de la chaine alimentaire.)	
19	T	Congruent	There are more organisms at the base of a food chain than at the top.<br>(Il y a plus d'organismes à la base d'une chaîne alimentaire qu'au sommet.)	The upper links of the food chain accumulate energy. [46]
19'	F	Incongruent	There are more organisms at the base of a food chain than at the top.<br>(Il y a plus d'énergie au sommet d'une chaîne alimentaire qu'à sa base.)	
20	T	Congruent	Biodegradable substances are not radioactive.<br>(Les substances biodégradables ne sont pas radioactives.)	Biodegradable substances cannot pollute. [47]
20'	F	Incongruent	Biodegradable substances are not polluting.<br>(Les substances biodégradables ne sont pas polluantes.)	

Pair #	Scientific Value	Congruency	Statement	Naïve Ideas
21	F	Congruent	When decomposing, matter is created.<br>(Lors de la décomposition, de la matière se crée.)	Decomposition is the disappearance of matter. [48]
21’	F	Incongruent	When decomposing, matter disappears.<br>(Lors de la décomposition, de la matière disparait.)	
22	T	Congruent	A synthetic substance can pollute.<br>(Une substance synthétique peut être polluante.)	Natural substances cannot pollute. [9,47]
22’	T	Incongruent	A natural substance can pollute.<br>(Une substance naturelle peut être polluante.)	
23	F	Congruent	Stones compete for the resources they need.<br>(Les pierres compétitionnent pour les ressources dont elles ont besoin.)	Plants do not compete to get access to resources. [49]
23’	T	Incongruent	Plants compete for the resources they need.<br>(Les plantes compétitionnent pour les ressources dont elles ont besoin.)	
24	F	Congruent	Apes descend from humans.<br>(Le singe descend de l’humain.)	Species evolve towards a more advanced form like humans. [50]
24’	F	Incongruent	Humans descend from apes.<br>(L’humain descend du singe.)	
25	F	Congruent	The first humans lived at the same time as mammoths.<br>(Les premiers humains vivaient en même temps que les mammouths.)	Humans and dinosaurs coexisted. [51]
25’	T	Incongruent	The first humans lived at the same time as dinosaurs.<br>(Les premiers humains vivaient en même temps que les dinosaures.)	
26	F	Congruent	Stones have DNA.<br>(Les roches ont de l’ADN.)	Not all fungi have DNA. [52]
26’	T	Incongruent	Fungi have DNA.<br>(Les champignons ont de l’ADN.)	
27	T	Congruent	An adult has more cells than an embryo.<br>(Un adulte possède davantage de cellules qu'un embryon.)	When growing, it is the cells that grow. [53]
27’	F	Incongruent	An adult has bigger cells than a child.<br>(Un adulte possède de plus grosses cellules qu'un enfant.)	
28	F	Congruent	Blue-eyed parents can have brown-eyed children.[i]<br>(Des parents aux yeux bleus peuvent avoir des enfants aux yeux bruns.)	A child receives a single copy of each gene. [27]
28’	T	Incongruent	Brown-eyed parents can have blue-eyed children.<br>(Des parents aux yeux bruns peuvent avoir des enfants aux yeux bleus.)	
29	F	Congruent	The blood is oxygenated in the bones.<br>(Le sang est réoxygéné dans les os.)	Blood and oxygen mix in the heart. [54]
29’	F	Incongruent	The blood is oxygenated in the heart.<br>(Le sang est réoxygéné dans le cœur.)	
30	F	Congruent	Arterial blood is rather blue.<br>(Le sang dans les artères est plutôt bleu.)	Venial blood is rather blue. [54]
30’	F	Incongruent	Venial blood is rather blue.<br>(Le sang dans les veines est plutôt bleu.)	

[i]: In rare cases, blue-eyed parents can give birth to a brown-eyed child [55]. However, as this is an infrequent event, the idea that brown-eyed parents can not have blue-eyed children is accepted in many educational and scientific publications [56].

Biology

Pair #	Scientific Value	Congruency	Statement	Naïve Ideas
31	T	Congruent	The heart pumps blood.<br>(Le cœur pompe du sang.)	The heart manufactures blood. [54]
31’	F	Incongruent	The heart manufactures blood.<br>(Le cœur fabrique du sang.)	
32	T	Congruent	The heart circulates the blood.<br>(Le cœur fait circuler le sang.)	The heart cleanses the blood. [54]
32’	F	Incongruent	The heart filters the blood.<br>(Le cœur filtre le sang.)	

Summary (biology statements)

Condition	Congruent		Incongruent	
Scientific response	True	False	True	False
	15	17	17	15

Summary (all statements)

Condition	Congruent		Incongruent	
Scientific response	True	False	True	False
	31	33	33	31

References

1. Hickey, R., & Schibeci, R. A. The attraction of magnetism. *Physics Education*, **34** 383–388 (1999).
2. Garnett, P. J., & Treagust, D. F. Conceptual difficulties experienced by senior high school students of electrochemistry: Electric circuits and oxidation-reduction equations. *Journal of Research in Science Teaching*, **29** 121–142 (1992).
3. AAAS. Item EG064001: Both a piece of metal that feels hot and a piece of metal that feels cold have thermal energy. http://assessment.aaas.org/items/0//EG064001#/0 (2017).
4. Williamson, K. E., & Willoughby, S. Student Understanding of Gravity in Introductory College Astronomy. *Astronomy Education Review*, DOI: 10.3847/AER2011025 (2012).
5. Trouille, L. E., Coble, K., Cochran, G. L., Bailey, J. M., Camarillo, C. T., Nickerson, M. D., & Cominsky, L. R. Investigating student ideas about cosmology III: Big bang theory, expansion, age, and history of the universe. *Astronomy Education Review*, https://doi.org/10.3847/AER2013016 (2013).
6. Driver, R., Squires, A., Rushworth, P., & Wood-Robinson, V. Making Sense of Secondary Science: Research into children's ideas. *Routledge* (2015).
7. Stead, K., & Osborne, R. *Gravity*. LISP Working Paper 20 University of Waikato. https://www.stem.org.uk/system/files/elibrary-resources/2018/12/BEST_PES_1_2_Key%20concept_Gravity%20-%20Teacher%20Notes.pdf (1980).
8. Baxter, J. Children's understanding of familiar astronomical events. *International Journal of Science Education*, **11** 502–513. (1989).
9. Sadler, P. M., Coyle, H., Miller, J. L., Cook-Smith, N., Dussault, M., & Gould, R. R. The Astronomy and Space Science Concept Inventory: Development and Validation of Assessment Instruments Aligned with the K–12 National Science Standards. *Astronomy Education Review*, **8** (2010).
10. Vosniadou, S. Capturing and modeling the process of conceptual change. *Learning and Instruction*, **4** 45–69 (1994).
11. Dunlop, J. How children observe the universe. *Publications of the Astronomical Society of Australia*, **17** 194 (2000).
12. Johnson, P. Children's understanding of changes of state involving the gas state, Part 1: Boiling water and the particle theory. *International Journal of Science Education*, **20** 567–583 (1998).
13. Osborne, R. J., & Cosgrove, M. M. Children's conceptions of the changes of state of water. *Journal of Research in Science Teaching*, **9** 825–838 (1983).
14. Coştu, B., Ayas, A., & Niaz, M. Promoting conceptual change in first year students' understanding of evaporation. *Chemistry Education Research and Practice*, **11** 5 (2010).
15. Durmuş, J., & Bayraktar, Ş. Effects of Conceptual Change Texts and Laboratory Experiments on Fourth Grade Students' Understanding of Matter and Change Concepts. *Journal of Science Education and Technology*, **19** 498–504 (2010).
16. Sadler, P., Coyle, H., Cook-Smith, N., & Miller, J. Misconceptions-oriented standards-based assessment resources for teachers (MOSART). *Cambridge, MA: Harvard College* (2007).

17. Eshach, H., & Schwartz, J. L. Sound Stuff? Naïve materialism in middle-school students' conceptions of sound. *International Journal of Science Education*, **28** 733–764 (2006).
18. Hewson, M. G. A. B. The acquisition of scientific knowledge: Analysis and representation of student conceptions concerning density. *Science Education*, **70** 159–170 (1986).
19. Gonen, S. A study on student teachers' Misconceptions and scientifically acceptable conceptions about mass and gravity. *Journal of Science Education and Technology*, **17** 70–81 (2008).
20. AAAS. Item AM046006: During a hot day in the summer, the spaces between the concrete sections of a sidewalk get narrower because the concrete sections expand. http://assessment.aaas.org/items/AM046006#/1 (2017).
21. Herrmann-Abell, C. F., & DeBoer, G. E. Probing Middle School Students' Knowledge of Thermal Expansion and Contraction through Content-Aligned Assessment. *National Association for Research in Science Teaching,* http://www.project2061.org/publications/2061Connections/2007/media/thermal_expansion_and_contraction_poster.pdf (2007).
22. Sneider, C., Bar, V., & Kavanagh, C. Learning about Gravity I. Free Fall: A Guide for Teachers and Curriculum Developers. *Astronomy Education Review* **5** 21–52 (2006).
23. AAAS. Item EG025001: In order to know which of two objects is moving faster, you need to know the weight (mass) of each object in addition to the motion energy. http://assessment.aaas.org/items/EG025001#/1 (2017).
24. Kesidou, S., & Duit, R. Students' conceptions of the second law of thermodynamics—an interpretive study. *Journal of Research in Science Teaching*, **30** 85–106 (1993).
25. AAAS. Item NG045002: Both a light bulb and an ice cream cone radiate energy because all objects radiate energy. Retrieved from http://assessment.aaas.org/misconceptions/NGM032 (2017).
26. Zeilik, M., Schau, C., & Mattern, N. Misconceptions and their change in university-level astronomy courses. *The Physics Teacher*, **36** 104–107 (1998).
27. Shtulman, A., & Valcarcel, J. Scientific knowledge suppresses but does not supplant earlier intuitions. *Cognition*, **124** 209–215 (2012).
28. Tatar, E. Prospective primary school teachers' misconceptions about states of matter. *Educational Research and Reviews*, **6** 197–200 (2011).
29. Palmer, D. Students' alternative conceptions and scientifically acceptable conceptions about gravity. *International Journal of Science Education*, **23** 691–706 (2001).
30. Holding, B. Investigation of schoolchildren's understanding of the process of dissolving with special reference to the conservation of mass and the development of atomistic ideas'. *University of Leeds,* http://etheses.whiterose.ac.uk/421/1/uk_bl_ethos_391600.pdf (1987).
31. Novick, S., & Nussbaum, J. Pupils' understanding of the particulate nature of matter: A cross-Age study. *Science Education*, **65** 187–196 (1981).
32. Trowbridge, J. E., & Mintzes, J. J. Alternative conceptions in animal classification: A cross-age study. *Journal of Research in Science Teaching*, **25** 547–571 (1988).
33. Kubiatko, M., & Prokop, P. Pupils' misconceptions about mammals. *Journal of Baltic Science Education*, **6** 5–15 (2007).

34. Thompson, F., & Logue, S. An exploration of common student misconceptions in science. *International Education Journal*, **7** 553–559 (2006).
35. Braund, M. Trends in children's concepts of vertebrate and invertebrate. *Journal of Biological Education*, **32** 112–118 (1998).
36. Sadler, P. M., Coyle, H., Smith, N. C., Miller, J., Mintzes, J., Tanner, K., & Murray, J. Assessing the life science knowledge of students and teachers represented by the K–8 National Science Standards. *CBE-Life Sciences Education*, **12** 553–575 (2013).
37. Dreyfus, A., & Jungwirth, E. The pupil and the living cell: a taxonomy of dysfunctional ideas about an abstract idea. *Journal of Biological Education*, **23** 49–55 (1989).
38. Banet, E., & Ayuso, E. Teaching genetics at secondary school: A strategy for teaching about the location of inheritance information. *Science Education*, **84** 313 (2000).
39. Yip, D. Y. Teachers' misconceptions of the circulatory system. *Journal of Biological Education*, **32** 207–215 (1998).
40. Özay, E., & Öztaş, H. Secondary students' interpretations of photosynthesis and plant nutrition. *Journal of Biological Education*, **37** 68–70 (2003).
41. Treagust, D. F., & Haslam, F. Evaluating Secondary Students' Misconceptions of Photosynthesis and Respiration in Plants Using a Two-Tier Diagnostic Instrument. *Annual Meeting of the National Association for Research in Science Teaching* https://files.eric.ed.gov/fulltext/ED283713.pdf (1986).
42. Treagust, D. F. Development and use of diagnostic tests to evaluate students' misconceptions in science. *International Journal of Science Education*, **10** 159–169 (1988).
43. Gayford, C. G. Some aspects of the problems of teaching about energy in school biology. *European Journal of Science Education*, ***8*** 443–450 (1986).
44. Tamir, P., Gal-Choppin, R., & Nussinovitz, R. How do intermediate and junior high school students conceptualize living and nonliving? *Journal of Research in Science Teaching*, **18** 241–248 (1981).
45. Gallegos, L., Jerezano, M. E., & Flores, F. Preconceptions and relations used by children in the construction of food chains. *Journal of Research in Science Teaching*, **31** 259–272 (1994).
46. Adeniyi, E. Misconceptions of selected ecological concepts held by some Nigerian students. *Journal of Biological Education*, **19** 311–316 (1985).
47. Brody, M. J. Student science knowledge related to ecological crises. *International Journal of Science Education*, **16** 421–435 (1994).
48. Leach, J., Driver, R., Scott, P., & Wood-Robinson, C. Children's ideas about ecology 2: ideas found in children aged 5-16 about the cycling of matter. *International Journal of Science Education*, **18** 19–34 (1996).
49. AAAS. Item IE074002: Plants compete for water, space, and light when those resources are limited. http://assessment.aaas.org/items/IE074002#/1 (2017).
50. Heddy, B. C., & Sinatra, G. M. Transforming Misconceptions: Using Transformative Experience to Promote Positive Affect and Conceptual Change in Students Learning About Biological Evolution. *Science Education*, **97** 723–744 (2013).
51. Conseil de la science et de la technologie. La culture scientifique et technique au Québec: Bilan. *Gouvernement du Québec*. https://www.sciencepourtous.qc.ca/wp-content/uploads/2012/06/CSTBilan2002.pdf (2002).

52. Mills Shaw, K. R., Van Horne, K., Zhang, H., & Boughman, J. Essay Contest Reveals Misconceptions of High School Students in Genetics Content. *Genetics*, **178** 1157–1168 (2008).
53. AAAS. Item CE119002: The difference in size between young children and fully grown adults can be explained by the repetitive process of cell growth and division. http://assessment.aaas.org/items/CE119002#/1(2017).
54. Arnaudin, M. W., & Mintzes, J. J. Students' alternative conceptions of the human circulatory system: A cross-age study. *Science Education*, **69** 721–733. (1985).
55. Starr, B. How Blue Eyed Parents Can Have Brown Eyed Children. http://genetics.thetech.org/how-blue-eyed-parents-can-have-brown-eyed-children (2013).
56. The Tech Museum of Innovation. What Color Eyes will your Children Have? http://genetics.thetech.org/online-exhibits/what-color-eyes-will-your-children-have (2013).

Supplementary Discussion

Main Effect of Expertise

Expertise-Dependent Modulation of Brain Activity

For the sake of completeness, we examined which regions were activated by the effect of EXPERTISE when congruency is averaged across all levels (Fig.1, Table 1). We explored cortical sites where brain responses are modulated by the level of expertise and, consequently, which activations are higher for physics statements compared to biology statements, or conversely. The analysis of variance identified an extensive set of areas associated with EXPERTISE involving the bilateral inferior temporal regions, bilateral inferior parietal lobules, right supramarginal gyrus and left MFG. Higher BOLD response in all clusters was found for statements in physics (advanced level of expertise) compared to biology (basic level of expertise). Additional areas are identified by t-contrasts and reveal that physics, more than biology, activated the bilateral IFG, bilateral precentral gyrus, and the right MFG, whilst biology, more than physics, activated the bilateral temporal poles, left temporal gyrus, left precentral gyrus, left superior frontal gyrus and right angular gyrus more than advanced expertise trials.

Table 1. Significant activation clusters for the main effect of EXPERTISE and the subsequent t-contrasts between physics (advanced level of expertise) and biology (basic level of expertise).

	MNI peak coordinate			Main effect of EXPERTISE			Physics > Biology			Biology > Physics		
Brain areas	x	y	z	k	F	$p_{FWE-corr}$	k	t	p_{uncorr}	k	t	p_{uncorr}
**Frontal Lobe**												
L Middle frontal gyrus	-24	6	60	161	24.47	.007	218	5.54	< .001	-	-	n. s.
L Inferior frontal gyrus (opercular), Precentral gyrus	-51	9	24	-	-	n. s.	251	4.85	< .0001	-	-	n. s.
R Inferior frontal gyrus (opercular), Middle frontal gyrus, Precentral gyrus	51	12	21	-	-	n. s.	113	4.05	< .0001	-	-	n. s.
L Superior frontal gyrus, medial	-9	51	36	-	-	n. s.	-	-	n. s.	47	4.98	< .0001
**Parietal Lobe**												
L Inferior parietal gyrus, Middle occipital gyrus	-57	-45	54	1551	57.00	< .001	1835	8.45	< .0001	-	-	n. s.
R Supramarginal gyrus, Inferior parietal gyrus	60	-30	48	305	34.25	< .001	1468* -	6.34	< .0001	-	-	n. s.
R Angular gyrus	51	-63	39	-	-	n. s.		-	n. s.	103	4.25	<.0001
**Temporal Lobe**												
L Inferior temporal gyrus	-51	-54	-3	384	98.94	< .001	403	9.45	< .0001	-	-	n. s.
R Inferior temporal gyrus	54	-51	-9	154	43.03	.027	155	6.50	< .0001	-	-	n. s.
L Temporal pole (middle) temporal gyrus, Precentral gyrus	-45	9	-30	-	-	n. s.	-	-	n. s.	133	5.46	< .0001
R Temporal pole (middle), Temporal pole (superior)	48	9	-30	-	-	n. s.	-	-	n. s.	82	4.22	.001
**Occipital Lobe**												
R Middle occipital gyrus, Middle occipital gyrus, Inferior parietal gyrus	39	-81	33	721	38.89	<.001	1468*	7.25	<.0001	-	-	n. s.

Note: Coordinates are reported in MNI space as given by SPM8, main effect at $p_{FWE\text{-}CORRECTED} < .05$ and t-contrasts at $p_{UNCORRECTED} < .005$, expected voxels per cluster k = 11 for main effect and k = 14 for t-contrasts, second-level analysis (random effect analysis, full factorial and t-tests). Coordinates are reported in MNI space as given by SPM8. Anatomical labels are based on the AAL (automated anatomical labeling) atlas [1]. The first label represents the location of the peak activation, additional labels denote submaxima if located in a different brain region. L = left; R = right. *One cluster included both R Supramarginal gyrus and R Middle occipital gyrus.

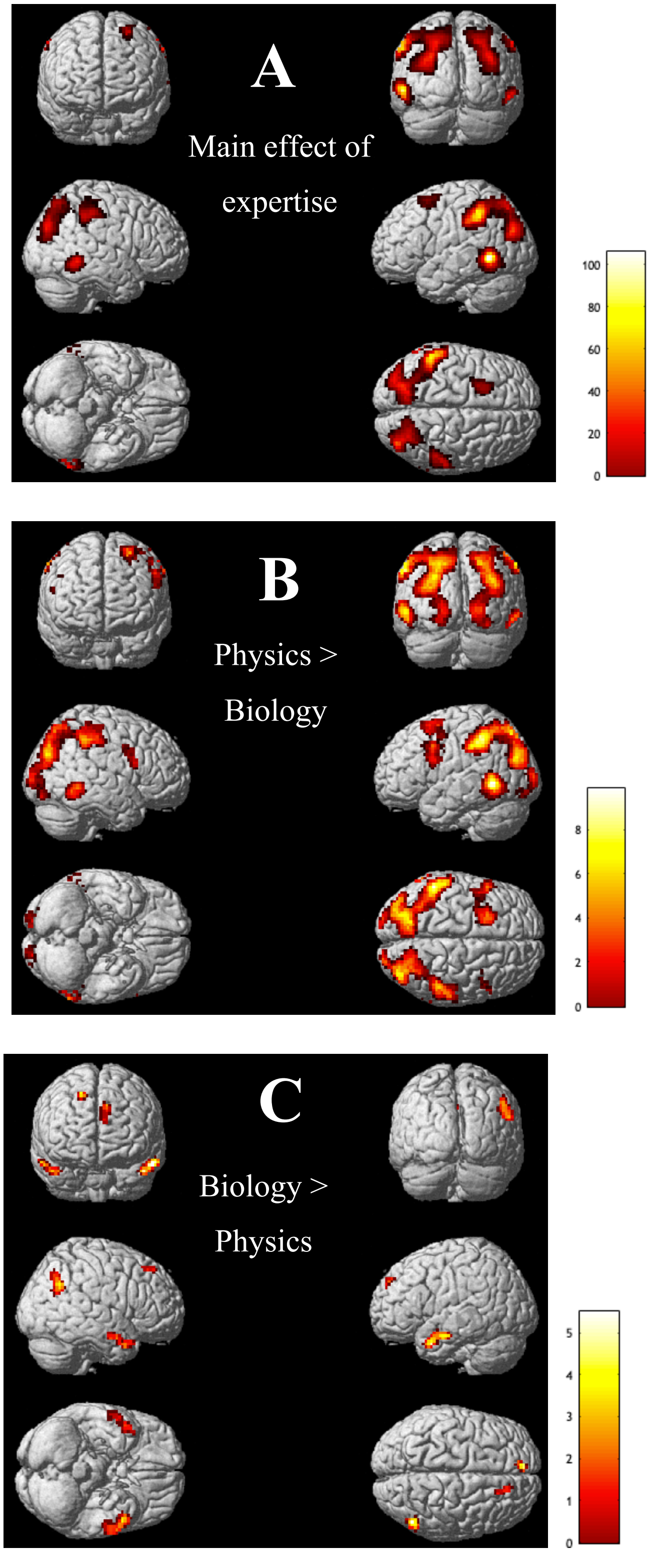

Fig. 1 Activation clusters for the main effects EXPERTISE (A) as well as the subsequent t-contrasts between physics > biology (B); biology > physics (C); Clusters are presented at $p_{UNCORRECTED} < .005$ and are depicted in the standard single-subject volume-rendered brain implemented in SPM8. L =left; R= right

Brain-Based Mechanisms of Scientific Expertise

The ANOVA yielded expertise-related activation differences in a network that comprised bilateral inferior temporal, bilateral inferior parietal (intraparietal), right supramarginal and left superior frontal gyrus sites. This network was activated by all physics statements, congruent and incongruent, but remained silent to biology statements. Those results are analogous to findings comparing brain activity of advanced expert mathematicians reflecting on mathematical and nonmathematical statements [2]. In fact, the existing literature suggests that the network we identified engages in a variety of reasoning processes that might lie at the core of thinking in physics, while likely also contributing to other forms of reasoning or problem solving. Our results coincide with regions previously associated with "multiple-demand" system [3] active in many effortful problem-solving tasks [4]. Some have suggested that these regions form a "general problem solving" network active in all effortful cognitive tasks [5], but these models are put into questions and our results also call them into question. The main argument being that, similar to Amalric & Dehaene [2] who compared mathematical and non-mathematical reasoning, we found no activation of this network during equivalently difficult assessment of the scientific value of biology statements.

Conversely, statements in biology for which participants possessed basic expertise activated areas that are distinctive to language, memory, and attention: bilateral anterior temporal, bilateral superior frontal and right angular regions. The anterior temporal region makes a crucial contribution to semantic cognition [6] while the superior frontal gyrus is involved in higher levels of working memory processing including monitoring and manipulation [7,8]. An abundant literature reveals that the angular gyrus is implicated in numerous tasks and processes [9]. The right angular gyrus is found to be strongly involved during the inhibition of the inappropriate response across a variety of go/no-go tasks [10,11]. It has also been shown that attentional reorienting originates in the right angular gyrus thanks to its causal role in using task history to update attentional selection [12]. In sum, the angular gyrus may play a role in maintaining attention [13] as well as in inhibitory control [10].

All things considered, the present results suggest that physicists evaluating physics statements might rely on specific brain networks, whereas physicists evaluating biology statements might be more strongly associated with general semantic knowledge as reflected by temporal activation patterns. However, we should interpret these results keeping in mind that statements in biology and physics despite being derived from what is believed to be equivalently persistent naïve ideas, were matched for concepts, complexity, word count, and syllable count within domains, but not when comparing them with each other.

Regions of Interest Analysis

In addition to whole-brain analysis, we conducted full factorial design analysis using a regions of interest (ROI) to model one main effect (CONGRUENCY) and one interaction effect (EXPERTISE and CONGRUENCY) to highlight differences in activation patterns between within-subject factors [14]. We conducted the analysis across the frontal lobe and anterior cingulate cortex using Automated Anatomical Labeling (AAL) atlas [1]. Similar to the whole-brain analysis, all brain activations resulting from the ANOVA are reported at the familywise error (FWE) corrected threshold $p_{FWE\text{-}CORRECTED}$ <.05 across the whole brain, using a primary voxelwise threshold of $p_{UNCORRECTED}$ < .005.

Congruency-Dependent Modulation of Brain Activity

Table 1. Significant activation clusters for the main effect CONGRUENCY and the subsequent t-contrasts between congruent and incongruent statements.

Brain areas	MNI peak coordinate			Main effect of CONGRUENCY			Incongruent > Congruent			Congruent > Incongruent		
	x	y	z	k	F	p FWE-corr	k	t	p uncorr	k	t	p uncorr
**Frontal Lobe**												
L Inferior frontal gyrus (orbital), R Inferior frontal gyrus (orbital), Bilateral Anterior cingulate gyrus, L Anterior middle frontal gyrus, R Frontal Pole, L Middle Frontal gyrus	-21	24	-9	238	27.34	.001	116	4.90	< .001	-	-	n. s.
R Supplementary motor area (lateral and medial), L Supplementary motor area (lateral and medial)	3	27	48	206	21.33	.003	147	4.46	< .0001	-	-	n. s.
L Supplementary motor area	-51	15	42	-	-	-	49	3.75	< .0001	-	-	n. s.

Note: Coordinates are reported in MNI space as given by SPM8, main effect at $p_{FWE\text{-}CORRECTED}$ < .05 and t-contrasts at $p_{UNCORRECTED}$< .005, expected voxels per cluster k = 11 for main effect and k = 14 for t-contrasts, second-level analysis (random effect analysis, full factorial and t-tests). Coordinates are reported in MNI space as given by SPM8. Anatomical labels are based on the AAL (automated anatomical labeling) atlas [1]. The first label represents the location of the peak activation, additional labels denote submaxima if located in a different brain region. L =left; R= right.

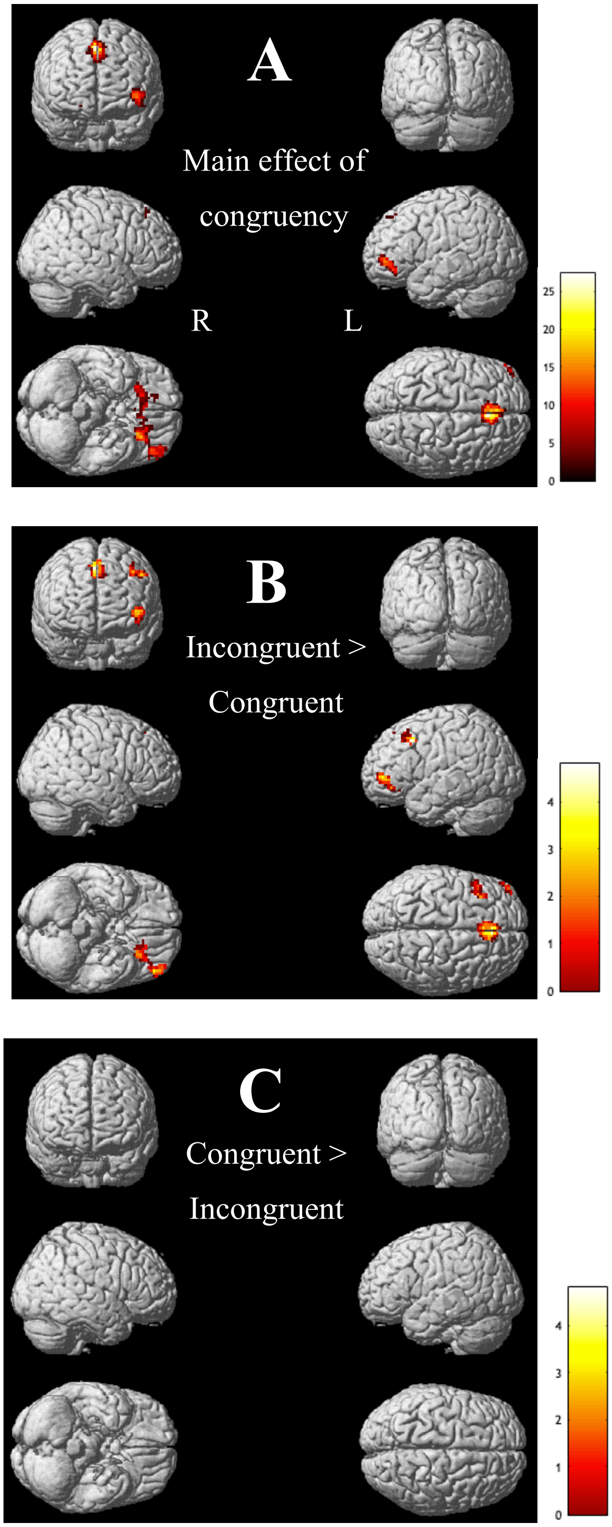

Fig. 2 Activation clusters for the main effect CONGRUENCY (A) as well as the subsequent t-contrasts between incongruent > congruent statements (B); and congruent statements > incongruent statements (C). Clusters are presented at $p_{UNCORRECTED} < .005$ and are depicted in the standard single-subject volume-rendered brain implemented in SPM8. L =left; R= right

“Expertise X Congruency” Dependent Modulation of Brain Activity

No significant activation cluster for the interaction effect between EXPERTISE and CONGRUENCY was found at familywise error (FWE) corrected threshold $p_{FWE\text{-}CORRECTED} < .05$ using a primary voxelwise threshold of $p_{UNCORRECTED} < .005$ when masking with a ROI across the frontal lobe and anterior cingulate cortex using Automated Anatomical Labeling (AAL) atlas [1]. However, three significant activation clusters passed an uncorrected voxel-wise threshold of $p < .005$ (Table 2, Figure 2) in the bilateral anterior cingulate cortex (ACC) and left inferior frontal gyrus (IFG).

Table 2. Significant activation clusters for the interaction effect between EXPERTISE and CONGRUENCY.

Brain areas	MNI peak coordinate			EXPERTISE X CONGRUENCY		
	x	Y	z	k	F	p_{uncorr}
**Frontal Lobe**						
L Anterior cingulate cortex	-15	36	18	73	21.71	< .001
R Anterior cingulate cortex	12	39	24	57	16.22	< .001
L Inferior frontal gyrus (orbitalis)	-18	36	-6	30	3.57	< .001

Note: Coordinates are reported in MNI space as given by SPM8 at $p_{UNCORRECTED\ CLUSTER\text{-}WISE} < .005$, expected voxels per cluster k = 11, second-level analysis (random effect analysis, full factorial and t-tests). Coordinates are reported in MNI space as given by SPM8. Anatomical labels are based on the AAL (automated anatomical labeling) atlas [1]. L =left; R= right.

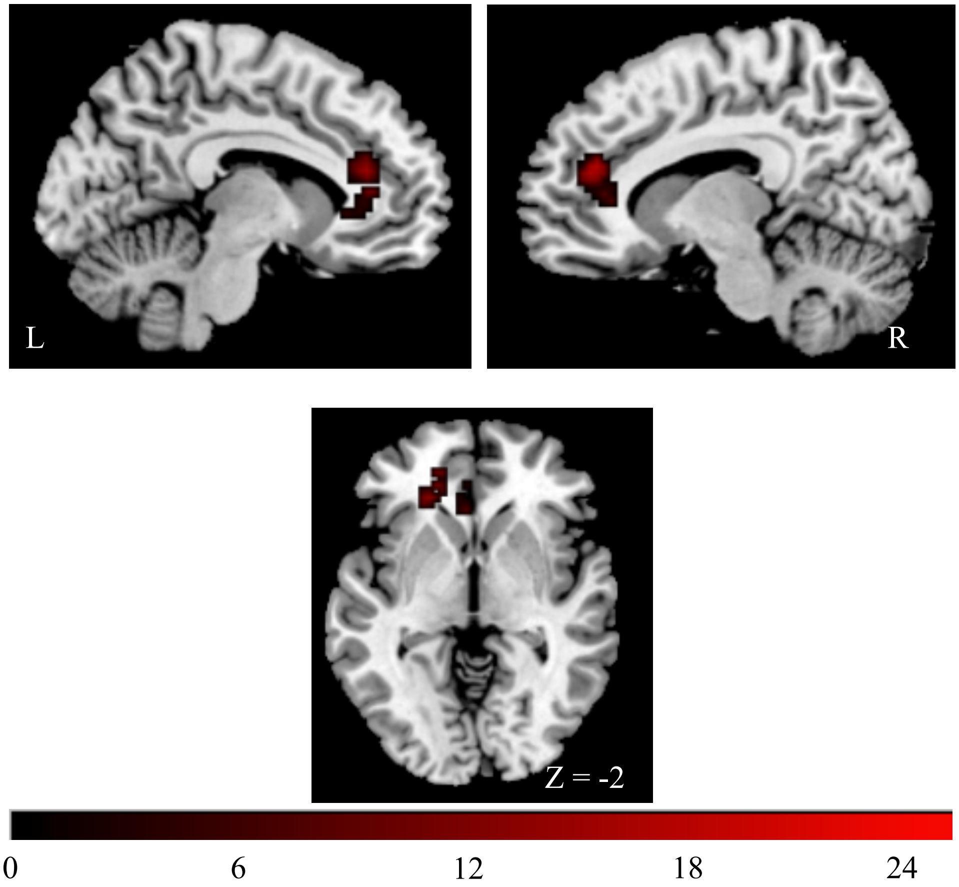

Fig. 3. Activation clusters for the interaction effect between EXPERTISE and CONGRUENCY depicted in the ch2better template in MRIcron [15] at $P_{\text{UNCORRECTED CLUSTER-WISE}} < .005$. L = left; R = right.

Then, we assessed the direction of the interaction effect. We examined which regions were more activated by incongruent or congruent statements for biology and physics independently. In physics (Table 3, Fig. 4), areas more activated for incongruent than congruent statements include bilateral supplementary motor area (SMA), bilateral middle frontal gyrus (MFG), right superior frontal gyrus (SFG) and left inferior frontal gyrus (IFG). No cluster is significantly more activated for congruent than incongruent statements.

Table 3. Activation clusters for t-contrasts between congruent and incongruent statements in physics

Brain areas	MNI peak coordinate					
	x	y	z	k	t	p uncorr
				INCONGRUENT > CONGRUENT		
**Frontal Lobe**						
L Supplementary motor area, R Supplementary motor area	-3	24	48	117	5.34	< .0001
L Supplementary motor area	-21	21	57	155	4.50	< .0001
R Middle frontal gyrus, R Superior frontal gyrus	33	21	54	125	4.31	< .0001
L Inferior frontal gyrus (triangularis)	-48	33	21	64	4.27	< .0001
L Middle frontal gyrus, L Middle frontal gyrus (orbitalis)	-42	54	3	55	4.20	< .0001
				CONGRUENT > INCONGRUENT		
-	-	-	-	-	-	n.s.

Note: Coordinates are reported in MNI space as given by SPM8, $P_{\text{UNCORRECTED CLUSTER-WISE}} < .005$, expected voxels per cluster k = 11, second-level analysis (random effect analysis, t-tests), MNI coordinates in mm. Anatomical labels are based on the AAL (automated anatomical labeling) atlas [1]. The first label represents the location of the peak activation, additional labels denote submaxima if located in a different brain region. L = left; R = right.

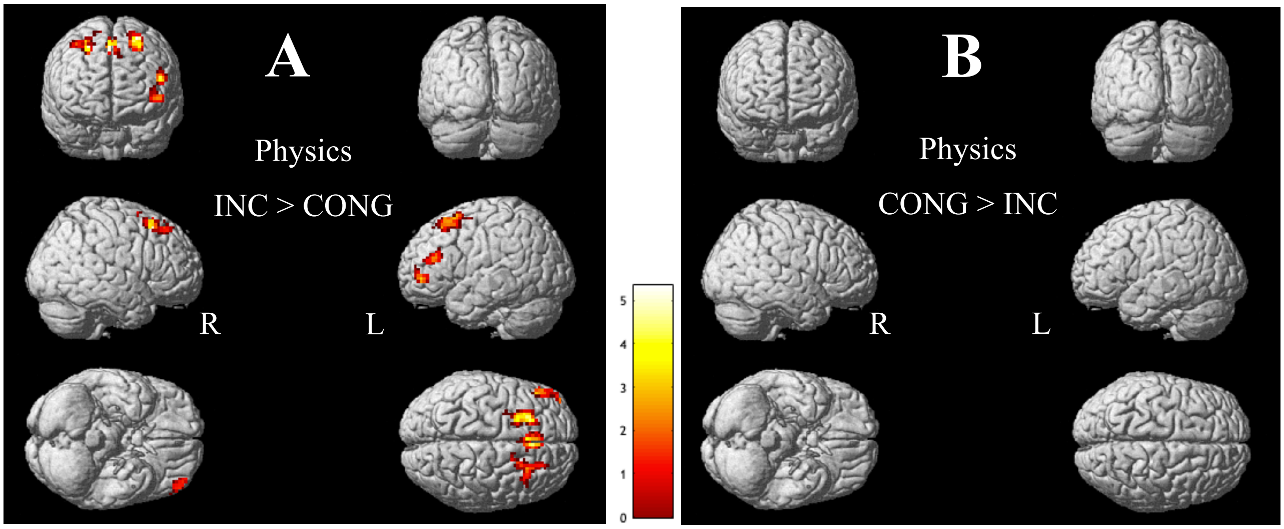

Fig. 4. Activation clusters for t-contrasts in physics: **a.** Incongruent statements > Congruent statement; **b.** Congruent statements > Incongruent statement. Clusters are presented at $P_{\text{UNCORRECTED CLUSTER-WISE}} < .005$ and are depicted in the standard single-subject volume-rendered brain implemented in SPM8. L = left; R = right.

Finally, we examined which regions were more activated by incongruent or congruent statements for biology. In physics (Table 4, Fig. 5), areas more activated for incongruent than congruent statements include bilateral superior frontal gyrus (SFG), bilateral inferior frontal gyrus, bilateral anterior cingulate cortex (ACC), right insula and left superior frontal gyrus. One cluster was more activated more by congruent statements compared to incongruent statements overlapping the left precentral gyrus and left postcentral gyrus.

Table 4. Activation clusters for t-contrasts between congruent and incongruent statements in biology

Brain areas	MNI peak coordinate					
	x	Y	z	k	t	P_{uncorr}
				INCONGRUENT > CONGRUENT		
**Frontal Lobe**						
L Superior frontal gyrus (orbitalis), L Inferior frontal gyrus (orbitalis), L Anterior cingulate cortex	-21	30	-12	67	4.53	< .0001
R Anterior cingulate cortex	12	39	24	40	3.97	< .0001
R Insula, R Inferior frontal gyrus (orbitalis), R Superior Frontal gyrus (orbitalis)	27	21	-15	30	3.95	< .0001
L Anterior cingulate gyrus, L Superior frontal gyrus (medial orbitalis)	-15	33	21	88	3.86	< .0001
				CONGRUENT > INCONGRUENT		
**Frontal Lobe**						
L Precentral gyrus, L Postcentral gyrus	-48	-6	42	57	3.87	< .0001

Note: Coordinates are reported in MNI space as given by SPM8, $P_{UNCORRECTED\ CLUSTER\text{-}WISE} < .005$, expected voxels per cluster k = 12, second-level analysis (random effect analysis, t-tests), MNI coordinates in mm. Anatomical labels are based on the AAL (automated anatomical labeling) atlas [1]. The first label represents the location of the peak activation, additional labels denote submaxima if located in a different brain region. L = left; R = right.

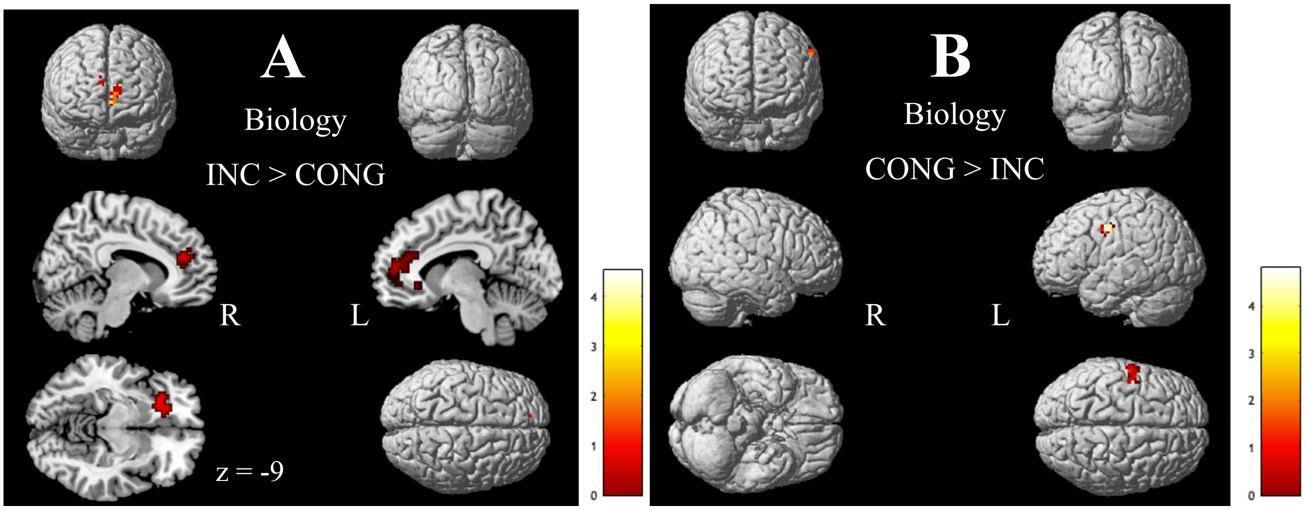

Fig. 5. Activation clusters for t-contrasts associated with statements in biology: **a.** Incongruent statements > Congruent statement, **b.** Congruent statements > Incongruent statement. Clusters are presented at $P_{\text{UNCORRECTED CLUSTER-WISE}}$ < .005 and 3D renders are depicted in the standard single-subject volume-rendered brain implemented in SPM8 while 2D renders are presented in the ch2better template in MRIcron [15]. L = left; R = right.

References

1. Tzourio-Mazoyer, N. et al. Automated anatomical labeling of activations in SPM using a macroscopic anatomical parcellation of the MNI MRI single-subject brain. *Neuroimage*, **15** 273-289 (2002).
2. Amalric, M., & Dehaene, S. Origins of the brain networks for advanced mathematics in expert mathematicians. *Proceedings of the National Academy of Sciences of the United States of America,* **113** 4909–4917 (2016).
3. Duncan, J. The multiple-demand (MD) system of the primate brain: mental programs for intelligent behaviour. *Trends in Cognitive Sciences,* **14** 172–179 (2010).
4. Fedorenko, E., Duncan, J., & Kanwisher, N. Broad domain generality in focal regions of frontal and parietal cortex. *Proceedings of the National Academy of Sciences,* **110** 16616–16621 (2013).
5. Hugdahl, K., Raichle, M. E., Mitra, A., & Specht, K. On the existence of a generalized non-specific task-dependent network. *Frontiers in Human Neuroscience,* https://doi.org/10.3389/fnhum.2015.00430 (2015).
6. Visser, M., Embleton, K. V., Jefferies, E., Parker, G. J., & Ralph, M. A. L. The inferior, anterior temporal lobes and semantic memory clarified: Novel evidence from distortion-corrected fMRI. *Neuropsychologia,* **48** 1689–1696 (2010).
7. Rottschy, C. et al. Modelling neural correlates of working memory: A coordinate-based meta-analysis. *NeuroImage,* **60** 830–846 (2012).
8. du Boisgueheneuc, F. et al. Functions of the left superior frontal gyrus in humans: A lesion study. *Brain,* **129** 3315–3328 (2006).
9. Seghier, M. L. The angular gyrus: multiple functions and multiple subdivisions. *The Neuroscientist*, **19** 43-61 (2013).
10. Nee, D., Wager, T., & Jonides, J. Interference resolution: Insights from a meta-analysis of neuroimaging tasks. *Cognitive, Affective, & Behavioral Neuroscience,* **7** 1–17 (2007).
11. Wager, T. D., Sylvester, C. Y. C., Lacey, S. C., Nee, D. E., Franklin, M., & Jonides, J. Common and unique components of response inhibition revealed by fMRI. *NeuroImage,* **27** 323–340 (2005).
12. Taylor, P. C. J., Muggleton, N. G., Kalla, R., Walsh, V., & Eimer, M. TMS of the right angular gyrus modulates priming of pop-out in visual search: Combined TMS-ERP evidence. *Journal of Neurophysiology* **106** 3001-3009 (2011).
13. Singh-Curry, V., & Husain, M. The functional role of the inferior parietal lobe in the dorsal and ventral stream dichotomy. *Neuropsychologia*, **47** 1434-1448 (2009).
14. Penny, W., & Henson, R. N. Analysis of Variance. *Statistical Parametric Mapping* (2006).
15. Rorden, C., Karnath, H. O., & Bonilha, L. Improving lesion-symptom mapping. Journal of Cognitive *Neuroscience,* **19** 1081-1088 (2007).
